# Axon and dendrite geography predict the specificity of synaptic connections in a functioning spinal cord network

**DOI:** 10.1186/1749-8104-2-17

**Published:** 2007-09-10

**Authors:** Wen-Chang Li, Tom Cooke, Bart Sautois, Stephen R Soffe, Roman Borisyuk, Alan Roberts

**Affiliations:** 1 School of Biological Sciences, University of Bristol, Woodland Road, Bristol BS8 1UG, UK; 2 Centre for Theoretical and Computational Neuroscience, University of Plymouth, Plymouth PL4 8AA, UK; 3 Department of Applied Mathematics and Computer Science, Ghent University, Krijgslaan 281-S9, B-9000 Ghent, Belgium

## Abstract

**Background:**

How specific are the synaptic connections formed as neuronal networks develop and can simple rules account for the formation of functioning circuits? These questions are assessed in the spinal circuits controlling swimming in hatchling frog tadpoles. This is possible because detailed information is now available on the identity and synaptic connections of the main types of neuron.

**Results:**

The probabilities of synapses between 7 types of identified spinal neuron were measured directly by making electrical recordings from 500 pairs of neurons. For the same neuron types, the dorso-ventral distributions of axons and dendrites were measured and then used to calculate the probabilities that axons would encounter particular dendrites and so potentially form synaptic connections. Surprisingly, synapses were found between all types of neuron but contact probabilities could be predicted simply by the anatomical overlap of their axons and dendrites. These results suggested that synapse formation may not require axons to recognise specific, correct dendrites. To test the plausibility of simpler hypotheses, we first made computational models that were able to generate longitudinal axon growth paths and reproduce the axon distribution patterns and synaptic contact probabilities found in the spinal cord. To test if probabilistic rules could produce functioning spinal networks, we then made realistic computational models of spinal cord neurons, giving them established cell-specific properties and connecting them into networks using the contact probabilities we had determined. A majority of these networks produced robust swimming activity.

**Conclusion:**

Simple factors such as morphogen gradients controlling dorso-ventral soma, dendrite and axon positions may sufficiently constrain the synaptic connections made between different types of neuron as the spinal cord first develops and allow functional networks to form. Our analysis implies that detailed cellular recognition between spinal neuron types may not be necessary for the reliable formation of functional networks to generate early behaviour like swimming.

## Background

**Figure 1:**
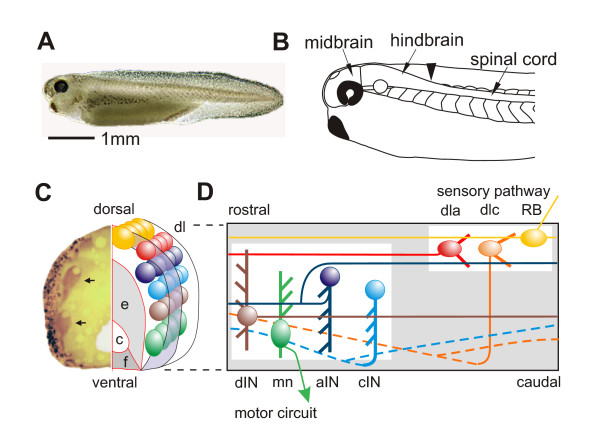
Hatchling *Xenopus *tadpole, nervous system and neurons. **(a) **Photograph of tadpole at stage 37/38. **(b) **The main parts of the CNS with arrowhead at hindbrain/spinal cord border. **(c) **Transverse section of the spinal cord with the left side stained to show glycine immunoreactive cell bodies (arrows) and axons (in the marginal zone). Diagrammatic right side shows the main regions: neural canal (c) bounded by ventral floor plate (f) and ependymal cell layer (e); lateral marginal zone of axons (mauve), layer of differentiated neuron cell bodies arranged in longitudinal columns (coloured circles) lying inside the marginal zone except in dorso-lateral (dl) and dorsal positions. **(d) **Diagrammatic view of the spinal cord seen from the left side, showing characteristic position and features of seven different neuron types. Each has a soma (solid ellipse), dendrites (thick lines) and axon(s) (thin lines). Commissural axons projecting on the opposite right side are dashed. See the text for details.

To function properly, nervous systems rely on highly specific synaptic connections between neurons. This specificity is achieved during development by many mechanisms, for example, correct neuronal specification and differentiation, axon path finding, cell recognition and synapse conditioning by neuronal activities. At the core of this, what are the rules that ensure that appropriate and specific synaptic connections are made as neuronal circuits develop? This is one of the most intensively studied areas of developmental neuroscience and has generated an extensive body of knowledge on the chemical cues that control the assembly of neuronal circuits in the central nervous system (CNS) [1-6]. The vertebrate spinal cord provides a simple example where chemical morphogens released from the dorsal roof plate (bone morphogenetic protein) and ventral floor plate (sonic hedgehog (Shh)) form dorso-ventral molecular gradients. These initially control the fate of differentiating neurons to establish a dorso-ventral series of longitudinal columns of distinct neurons on each side (Figure 1c) [7,8]. Once a cell has acquired a specific neuronal fate, the next step is to grow an axon from the neuron soma. The factors controlling the directions of outgrowth are beginning to be revealed [9]. Remarkably, the same morphogen gradients that control cell fates can also influence axon growth. For example, the Shh gradient can attract some axons to grow ventrally and cross to the opposite side [10]. After crossing, these axons are transformed and no longer attracted to the ventral floor plate [11-13]. They then turn to grow longitudinally [14], either towards the head or the tail, or they branch to grow in both directions. In all parts of the CNS such early patterns of growth by pioneer axons, controlled by chemical morphogens, lay down a basic scaffold of axon tracts that can be followed by later axons and in this way help to direct their growth [15]. Once the axons have grown to approximately the 'correct' area, they start to make connections (synapses) with the branched dendrites emerging from the cell bodies of other neurons.

We aim to answer two questions about the formation of synaptic connections. Our first question is: how accurate and specific are the synaptic connections formed during early stages of development within the CNS? Once axons have reached a suitable area to make synapses, cellular recognition processes [16] and activity-dependent mechanisms [17-20] may be needed to ensure that appropriate synaptic connections are made. However, our second question is: can simple factors, such as the broad geographical distributions of axons and dendrites, themselves generate sufficient specificity in synaptic connections to ensure the development of functional neuronal circuits?

To investigate the specificity of early synapse formation, we need to examine connections between identified neuron types in a functioning neuronal network. Very few vertebrate networks are simple enough to allow this; an exception is the developing spinal cord of the newly hatched clawed toad (*Xenopus laevis*) tadpole. Like the developing zebrafish [21,22], this spinal cord contains less than 2,000 neurons divided into very few types (approximately ten) yet it allows simple reflexes and swimming. In *Xenopus*, whole-cell recordings from pairs of spinal neurons under visual control have allowed us to build a remarkably full picture of the morphology, properties, synaptic connections and functions of the neurons and networks controlling swimming behaviour [23-28]. This detailed knowledge of the anatomy and function of different types of spinal neurons in developing *Xenopus *embryos provides a remarkable opportunity to use the whole-cell recording method to examine large numbers of synaptic connections between different types of identified spinal neuron to assess the specificity of the connections between them.

Our direct examination of synaptic connections between spinal neurons shows that connections are widespread and non-specific. We therefore examine the anatomy to see whether some very simple factors, like the different dorso-ventral distributions of the axons and dendrites of different neuron types, are sufficient to predict the connectivity found physiologically. We then use modelling to ask whether simple rules can reproduce longitudinal axon growth paths, and whether network models of the spinal circuits can produce swimming activity when synaptic connections are determined by simple probabilistic rules. Overall, our results show that it is possible that the first, pioneer neuronal networks formed in the spinal cord could be generated without specific neuron-to-neuron recognition mechanisms playing a necessary role in determining synaptic connectivity.

## Results

### Neuron types in the hatchling tadpole spinal cord

The two day old, hatchling *Xenopus *tadpole is 5 mm long (Figure 1a,b). The eyes are not yet functioning but the brain and spinal cord contain differentiated neurons. The spinal cord is a simple tube (approximately 0.1 mm diameter) with a central neural canal formed by ependymal cells and the ventral floor plate (Figure 1c). On each side lies a layer of nerve cells or neurons loosely organized into longitudinal columns. The neurons project processes into a superficial zone of longitudinal axons either directly or by first growing ventrally across the floor plate to the other side and then turning or branching longitudinally. As in all vertebrates, newly formed neurons are positioned in a dorsal to ventral sequence: sensory neurons; sensory interneurons; other interneurons; motoneurons. Unlike adult vertebrates, the young tadpole spinal cord has remarkably few types of spinal neuron, possibly less than ten. In this paper we consider seven types of spinal neuron involved in swimming (Figure 1d) with anatomy shown by dye filling and where the synapses made onto other spinal neurons have been defined by electrical recordings from pairs of individual neurons [29] (see also below). All synapses are made directly from longitudinal axons as they pass small processes emerging from the neurons called dendrites that protrude towards the side of the spinal cord.

### Evidence from recordings on synaptic connections between neuron types

To investigate the specificity of synaptic connections between the seven different types of spinal neuron, we used the whole-cell patch method to make current clamp recordings from over 500 pairs of neurons located 0.5 to 3 mm from the midbrain and usually recorded less than 0.3 mm apart. By injecting current into each neuron to evoke an action potential, we could see if a short-latency post-synaptic excitation or inhibition was present in the other neuron. After recording, the animals were fixed, the CNS removed and the anatomy of the recorded neurons revealed by neurobiotin staining. Only those pairs with clear anatomical identification and where the axon of at least one neuron was seen to have a possible contact point onto the dendrites of the other neuron were included in the analysis. Clearly, it was critical that we should know that the connections were monosynaptic. Synapses in the young tadpole can be unreliable, so standard tests for monosynaptic connections, involving high frequency following of presynatic spikes, were not possible. We therefore based our conclusions on measurements of latency combined with anatomy. The young tadpole has fine unmyelinated axons that conduct action potentials relatively slowly (approximately 0.3 m s^-1^) [30] and latencies depend on the distance between the neurons [28]. Most recordings were from pairs <0.3 mm apart and had latencies of <3 ms. Latencies up to 3.6 ms were found only with larger separations, up to 0.7 mm. These latency measurements are in complete accordance with our earlier studies of monosynaptic connections [31]. For known disynaptic pathways in the tadpole [25,26], typical latencies are at least 6 ms for equivalent separations, making it highly unlikely that the connections we report here were disynaptic. This direct evidence for connections was supported by observing synaptic potentials produced by stimulating sensory neurons in the skin or occurring during swimming.

### Rohon-Beard neuron synapses

**Figure 2:**
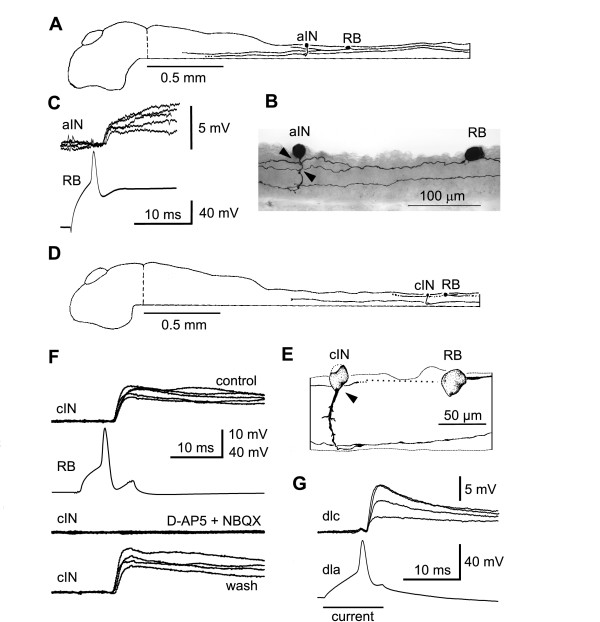
Recording synaptic connections. **(a-c) **RB sensory neuron excites an aIN: (a) side view of the isolated brain and spinal cord to show the location of both neurons and their axons; (b) anatomy of recorded neurons with possible synaptic contacts from RB axons onto aIN (arrowheads); (c) injection of current into RB evokes an action potential that leads to a short latency EPSP in the aIN (five traces overlapped). **(d-f) **RB excites a cIN: (d) location and (e) anatomy of RB and cIN pair; (f) current evoked RB action potentials lead to EPSPs (five traces overlapped) blocked reversibly by glutamate antagonists D-AP5 (25 *μ*M) + NBQX (2.5 *μ*M). **(g) **Current evoking an action potential in a dla produces short latency excitation (EPSPs) in a dlc (four traces overlapped).

Dorsal Rohon-Beard (RB) neurons are sensory, innervate the skin and respond to touch. Their central axons ascend and descend to excite other neurons by release of glutamate to activate α-amino-3-hydroxy-5-methyl-4-isoxazolepropionate (AMPAR) and *N*-methyl-D-aspartate receptors (NMDAR) [32,33,25]. In paired recordings where there is a direct synaptic connection, RB action potentials evoked by injected current lead to large excitatory postsynaptic potentials (EPSPs; 3.4–25.4 mV) at short and constant latencies (1.4–3.4 ms) [25,26]. Figure 2 shows examples of how synaptic connections were determined. In the first case, when the RB is stimulated, an ascending interneuron (aIN) is excited. In the second, the RB excites a commissural interneuron (cIN) and excitation can be blocked by glutamate receptor antagonists. Recordings from 132 pairs of neurons showed that the probability of finding synapses from RBs to dorsolateral commissural interneurons (dlcs) and dorsolateral ascending interneurons (dlas) is higher than from RBs to aINs and cINs (Table 1).

**Table 1:** Probabilities of synaptic connections found by paired recording and data from other tests to show presence/absence of connections

	**Post neuron**						
	
**Pre neuron**	**RB**	**dlc**	**dla**	**aIN**	**cIN**	**dIN**	**mn**
**RB **ipsi	0 (0/2)	0.63 (34/54)	0.35 (6/17)	0.13 (2/15)	0.09 (4/44)	+	+
**dlc **contra	-	0 + (0/1)	-	0.33 (2/6)	0.43 (18/42)	0.33 (1/3)	0.46 (6/13)
**dla **ipsi	0 (0/17)	0.18 (2/11)	-	0.25 (2/8)	0.08 (1/12)	0 (0/2)	-
**aIN **ipsi	0.07 (1/15)	[0.81] (17/21)	0.38 (3/8)	0.25 (4/16)	0.15 (6/39)	0.2 (2/10)	0.33 (1/3)
**cIN **contra	-	0 + (0/42)	0 +	0 ++ (0/13)	0.26 (9/35)	1 ++ (1/1)	0 ++ (0/3)
**dIN **ipsi	-	+	0 + (0/2)	++ (6/7)*	++ (6/7)*	++ (45/62)*	++ (27/32)*

Figures in parentheses give synapses found over number of pairs tested. For each neuron type, ipsi refers to synapses made on the same side and contra refers to synapses made on the opposite side. +, rare connections inferred from other experiments; ++, common connections but no quantitative data; *, connections frequent but preliminary recordings were used to select pairs of neurons that were connected, so connection probabilities are not meaningful. Square brackets indicate an artificially high value for aIN contacts.

The inaccessibility of more ventral descending interneurons (dINs) and motoneurons (mns) prevented paired recordings with RBs, so we electrically stimulated RB neurites in the skin. EPSPs with short and constant latencies (<6 ms), indicating direct connections, were found in 2/10 dINs and 1/12 mns.

### Dorsolateral commissural interneuron synapses

**Figure 3:**
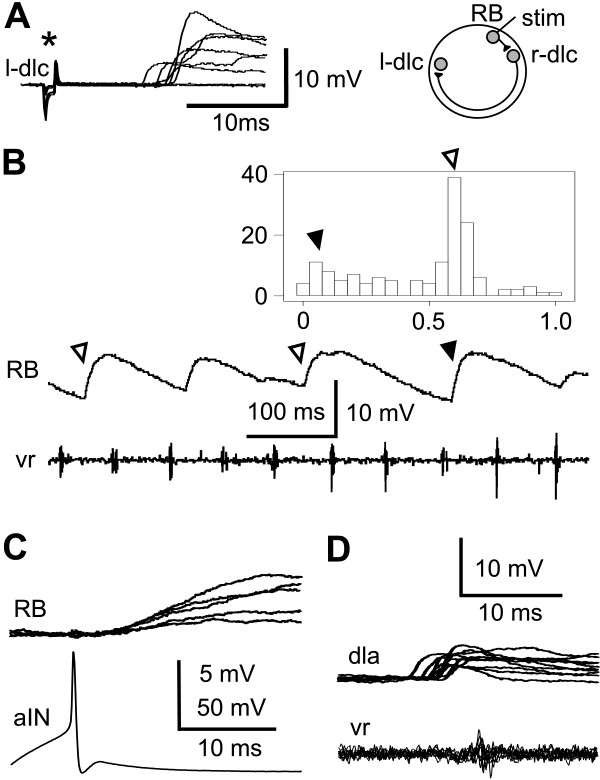
Unexpected synaptic connections. **(a) **In a left dlc (l-dlc) interneuron excitation is seen after variable delays as skin stimulation strength to the opposite right side increases (asterisk). The inset shows the probable pathway. **(b) **In a RB neuron, IPSPs (depolarising at resting membrane potential) occur during swimming, shown in a motor nerve recording (vr). Some IPSPs are mid-cycle (open arrowheads) and others are early-cycle (filled arrowhead). The histogram shows the phase distribution of 148 IPSPs in the swimming cycle. **(c) **Stimulating an aIN to fire an action potential leads directly to depolarising IPSPs at short latency in a RB neuron. **(d) **In a dla, fast on-cycle EPSPs, presumed to come from dINs, are seen on 77% of cycles during swimming.

Dlcs are sensory pathway interneurons excited by sensory RB neurons. They release glutamate to excite contralateral neurons via AMPARs and NMDARs. They mediate a flexion reflex and initiate swimming activity when the skin is stimulated [25]. Paired recordings showed that dlcs can directly excite all four types of neuron (aIN, cIN, dIN and mn; Table 1) that are active in swimming and called central pattern generator (CPG) neurons. Rather surprisingly, whole-cell recordings from 17 dlcs showed that 10 received EPSPs following contralateral skin stimulation (Figure 3a). The longer latencies of these EPSPs (7–14 ms) suggested that they were not direct but could originate from dlcs excited by skin stimulation on the opposite side of the body.

### Dorsolateral ascending interneuron synapses

Dlas are sensory pathway interneurons like dlcs that relay excitation from sensory RB neurons to more rostral ipsilateral CPG neurons [26]. Paired recordings and EPSP timing analyses showed that dlas could directly excite all types of CPG neurons (Table 1) [26]. In 2/11 paired recordings, dlas also excited dlcs (Figure 2g).

### Ascending interneuron synapses

AINs release glycine and have a broad dorsal-ventral distribution. They inhibit neurons on the same side in both *Xenopus *tadpole and developing zebrafish spinal cord [23,34]. Paired recordings made between aINs and other ipsilateral neurons showed that aINs directly inhibited all types of neurons (Table 1).

Because aINs are active during swimming, they produce inhibition in neurons on the same side early in each swimming cycle [23,28]. Early cycle inhibitory postsynaptic potentials (IPSPs) in RB neurons during swimming are very rare but were seen in 3/136 RB neurons (Figure 3b). This connection was confirmed in 1/15 paired recordings between aINs and RBs (Figure 3c). Since RB neurons do not usually have dendrites, these synapses may be onto presynaptic regions of synapses made by RB axons that would need to be close to the soma for any PSP to be recorded.

### Commissural interneuron synapses

CINs are a middle dorso-ventral group of glycinergic neurons that produce mid-cycle inhibition of CPG neurons on the opposite side of the spinal cord [35] to organize the alternation of activity between the two sides during swimming. Since they have been studied extensively [36], we made few paired recordings and cINs were only shown to produce direct contralateral unitary IPSPs in nine cINs and one dIN (Table 1). However, a consistent picture is revealed in recordings during swimming where reliable mid-cycle IPSPs/inhibitory postsynaptic current were seen in all types of CPG neurons (see figure 4 in [24]). They were also present in a small proportion of recordings from dlcs [28] and dlas [26], and in 1/146 RBs (Figure 3b).

**Figure 4:**
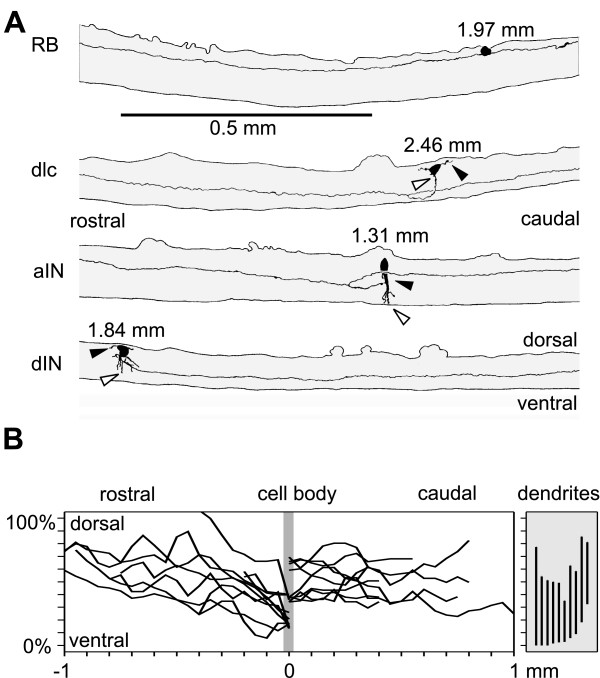
Dorso-ventral distribution of axons and dendrites. **(a) **Examples of neurobiotin filled neurons traced in lateral views of the spinal cord to show the dorso-ventral positions of the soma, dendrites and part of the axons. Dendrites emerge from the black soma, with the most ventral dendrite (open arrowhead) and most dorsal (black arrowhead) marked. Axons are on the same side as the soma except for dlcs where they cross ventrally then branch. Rostral to left, dorsal up. **(b) **Examples of axon trajectories of individual aINs (measured at 0.05 mm steps from the soma at 0 mm) and dorso-ventral extent of their dendrites (vertical lines at right).

### Descending interneuron synapses

DINs corelease glutamate and acetylcholine to excite other neurons via AMPAR, NMDAR and nicotinic acetylcholine receptors [31,27]. They provide ipsilateral excitation to CPG neurons during tadpole swimming [31,37]. In paired recordings, dINs were shown to directly excite all four types of CPG neurons, including other dINs (Table 1). Recordings in sensory pathway dlc and dla interneurons show that 9 of 43 dlcs and 1 of 2 dlas received weak on-cycle excitation (Figure 3d). The simplest explanation is that this excitation comes from dINs.

Overall, the results from paired recordings and other physiological recordings summarized in Table 1 reveal very widespread connectivity. Where evidence is available, neurons with dendrites (all except RBs) receive synapses from all other neuron types. This was unexpected and raised the possibility that the formation of synaptic connections in the developing spinal cord may be stochastic and not precisely determined by detailed processes of cell-to-cell recognition.

### Anatomical evidence on the dorso-ventral distribution of axons and dendrites

One alternative to specific cell-cell recognition mechanisms is that axons can chemically recognise neuronal dendrites and simply make synapses with any that they contact (in transmission electron microscope studies we have found very few axon-soma synapses; A Walford and A Roberts, unpublished). If this hypothesis is correct, the probability of contact will depend mainly on the dorso-ventral distribution of axons and dendrites, since axons run along the spinal cord, rarely branch, and make synapses directly onto dendrites that they pass. We have therefore examined these distributions for six spinal neuron types in the rostral spinal cord. Dlas form a small population [26] and there were not enough examples to include them in this analysis.

Neurons for anatomical analysis were selected where the soma was in the region where our electrical recordings were made (1 to 3 mm from the midbrain; Figure 1b). Spinal interneurons were filled individually with neurobiotin using sharp micropipettes inserted from the dorsal surface of the intact cord (Figure 4a; see also [24] and [38]). The axons are all relatively straight with maximum tortuosity (actual length/straight line distance) of 1.02 (n = 6). Figure 4b uses the aINs to illustrate the dorso-ventral distributions of axons and dendrites.

**Figure 5:**
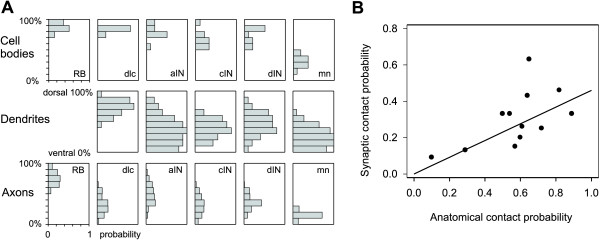
Axons, dendrites and synapse probabilities. **(a) **Histograms summarise dorso-ventral distribution of cell bodies, dendrites and axons of different neuron types in 10% bins where 0% is ventral and 100% dorsal edge of spinal cord. Distributions are expressed as the probability that a neuron will have a soma or dendrite in a particular dorso-ventral position. Axon distributions are expressed as the probability that a 50 *μ*m segment of the axon from each type of neuron will lie in a particular dorso-ventral position. **(b) **Plot of synapse probability from recordings versus contact probability from anatomy for cases in bold in Table 2. Highest point (RB-dlc) was omitted in calculating regression.

The dorso-ventral range of dendrites was determined from the positions of the most ventral and dorsal dendrite for each neuron (Figure 4a,b). This range will limit the number of axons contacted. We ignore the possibility that dendrites might be unevenly distributed within this range. The dendrite dorso-ventral ranges were summed for each neuron type, except RB neurons, which do not have dendrites. For each 10% dorso-ventral position bin (spinal cord diameter is approximately 100 *μ*m so bin width is approximately 10 *µ*m) in the 10 *μ*m thick marginal zone where dendrites and axons lie, we found the probability that an individual neuron of each type would have dendrites occupying that bin (Figure 5a). The dendrite distributions for neurons active during swimming (mns, aINs, cINs and dINs) were broad but all had a maximum just below the dorso-ventral midline (in the 30% or 40% bin) and fell away dorsally. In contrast, the dendrites of dlc sensory pathway interneurons had a maximum dorsally (in the 80% bin) and fell away ventrally.

We measured the dorso-ventral position of axons every 0.05 mm up to a maximum of 1 mm from the neuron soma. For each individual neuron, we pooled these measurements (discarding information about the distance from the soma and whether the axon was ascending or descending) and used them to calculate the probability of the axon occupying different dorso-ventral positions. These individual distributions were then averaged for all members of a type (Figure 5a; Table in Anatomy section of Additional file 1). The dorso-ventral axon distributions of some neurons are rather narrow. RB sensory neuron axons are dorsal (from 50% to 100%; maximum at 80%) while mns are ventral (from 10% to 30%; maximum at 20%). CINs, dINs and dlcs are all slightly biased towards ventral positions (from 10% to 60%) while inhibitory aINs have a broad axon distribution (10% to 90%; maximum at 40%).

Once the dorso-ventral distributions of axons and dendrites were established, 'contact' probabilities between axons and dendrites were calculated as follows for each pair of neuron types. The probabilities of individual axons or dendrites occupying a particular 10% dorso-ventral region were those plotted in Figure 5a. The probability that a particular pre-synaptic axon and post-synaptic dendrite would both occupy the same dorso-ventral region in the narrow marginal zone, and could therefore make contact, was simply the product of these probabilities. Overall contact probabilities between each type of neuron were then found by summing the separate probabilities for the ten dorso-ventral regions (Table 2). The contact probabilities range from 0.04 to 0.91 and relate intuitively to functions. They are higher for RB sensory neuron contacts onto sensory pathway dlcs (0.65) than onto other neurons like dINs (0.29); they are low for dlc contacts with each other (0.08) but higher onto the neurons activated after skin stimulation (0.54–0.89 for aINs, cINs, dINs and mns); they are quite high for contacts between neurons active during swimming (0.5–0.91 for aIN, cIN and dIN contacts to each other and to mns).

**Table 2:** Probabilities of synapses (from Table 1) and of potential 'contacts' between axons and dendrites for each neuron type

	**Dendrites**				
**Axons**	**dlc**	**ain**	**cin**	**din**	**mn**
**RB **ipsi					
Synapse	**0.63**	**0.13**	**0.09**	+ (0.13)	+(0.02)
Contact	0.65	0.29	0.10	0.29	0.04
**dlc **contra					
Synapse	0+ (0.04)	**0.33**	**0.43**	**0.33**	**0.46**
Contact	0.08	0.89	0.64	0.54	0.82
**aIN **ipsi					
Synapse	(0.13)	**0.25**	**0.15**	**0.2**	**0.33**
Contact	0.28	0.72	0.57	0.60	0.50
**cIN **contra					
Synapse	0+ (0.04)	0++ (0.40)	**0.26**	++ (0.24)	0++ (0.37)
Contact	0.08	0.88	0.61	0.52	0.80
**dIN **ipsi					
Synapse	+ (0.04)	*++ (0.42)	*++ (0.34)	*++ (0.29)	*++ (0.37)
Contact	0.08	0.91	0.73	0.64	0.80
**mn **ipsi					
Synapse	(0.00)	(0.41)	(0.18)	(0.10)	(0.45)
Contact	0.00	0.89	0.40	0.22	0.98

Synapse probabilities in bold are those from recordings based on more random sampling. Where there are no data from recordings, estimates of synaptic contact probabilities (in parentheses) are 46% of the anatomically estimated contact probabilities. For each neuron type, ipsi refers to synapses made on the same side and contra refers to synapses made on the opposite side. +, rare connections inferred from other experiments; ++, common connections but no quantitative data; *, connections frequent but preliminary recordings were used to select pairs of neurons that were connected, so connection probabilities are not meaningful.

When contact probabilities determined from anatomy were compared to synapse probabilities determined directly by electrical recording (Table 2), the two were significantly correlated for pairs where the neurons were randomly chosen for recording (bold entries in Table 2; Pearson correlation coefficient 0.593; *p *= 0.042). This significant relationship based on data from both anatomy and physiology (Figure 5b) was then used to predict the synaptic contact probability for cases with only anatomical data (Table 2). We first omitted data for contacts from RB to dlc neurons where the extensive rostro-caudal dendrites of dlc neurons are likely to result in a relatively high synaptic contact probability (see Discussion). The slope obtained by linear regression for the remaining points suggests that the probability of a real synaptic contact is around 46% of that predicted by anatomy.

We suggest on the basis of these results that during the formation of early synapses in the developing frog spinal cord, the different synapse probabilities found could depend simply on differences in the geographical distributions of axons and dendrites of different neuron types. These distributions could be sufficient to ensure, for example, that dorsal sensory RB axons synapsed mainly with dorsal sensory pathway interneurons rather than with more ventral neurons active during swimming (like mns).

### Modelling axonal growth and synaptic contact probabilities

Since axons grow a considerable distance along the spinal cord (often 1 to 2 mm in a 5 mm long animal) and can wander dorsal or ventral as they grow, their pattern of growth will have a strong influence on their potential to contact dendrites of different neuron types (Figure 4a). We concluded above that synaptic contacts may depend simply on dorso-ventral axon and dendrite distribution patterns. We therefore investigated whether a simple model, without any cell-cell recognition, could generate patterns of axon growth that would reproduce the observed axon distributions and, therefore, the synaptic contact probabilities. For simplicity, we assumed that dendrites are static and passive (see Discussion).

Our computational model starts from the point when axons start to grow longitudinally (Figures 1d and 4a). This point will be determined by the position of the soma and the initial behaviour of the axon. In the case of RB neurons, the axons grow directly from the soma towards the head and tail. In most other spinal neurons the axon first grows ventrally and then turns to grow longitudinally either on the same side or after crossing ventrally to the other side. We use the experimental observations to give us starting positions and initial growth angles of axons as well as their final lengths. A repetitive process of advancing the axon 1 *μ*m along its current growth angle and then modifying the growth angle is then applied until the predetermined rostrocaudal length of the axon is reached.

**Figure 6:**
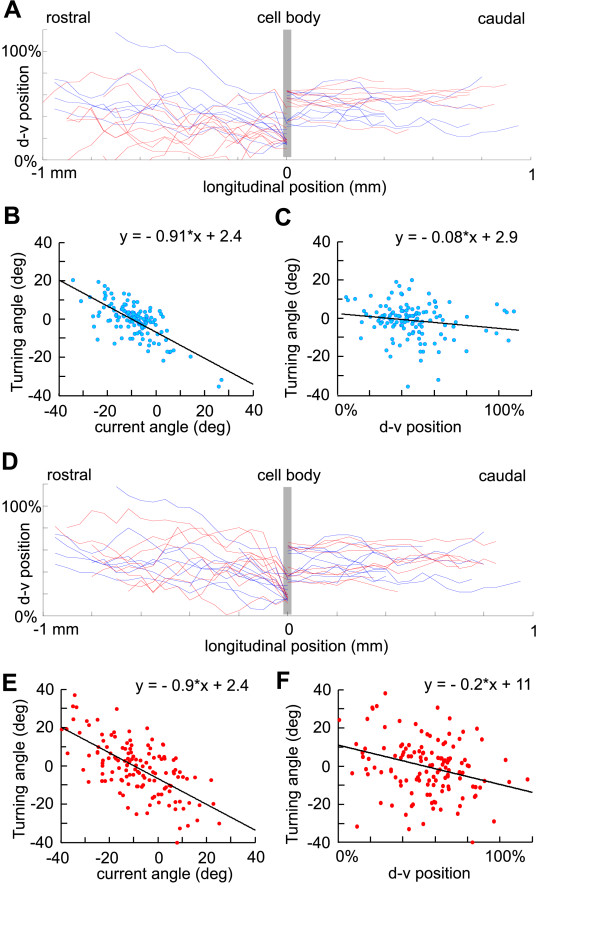
Modelling aIN axon growth and positional effects on axon turning angles. **(a) **aIN descending axons generated by a simple random growth model (red) fit the distribution of real descending axons (blue, to right) but model ascending axons do not match real ascending axons. **(b,c) **Real aIN ascending axon turning angles depend on the current growth angle and dorso-ventral (d-v) position. **(d) **In a model where growth angle depends on dorso-ventral position, generated aIN axons (red) match real axons (blue) closely. **(e,f) **Turning angles of modelled axons significantly match dependence of real axons on current angle and dorso-ventral position.

The current location and orientation of the tip of the axon (growth cone) are represented by three variables: x(t) rostrocaudal position, y(t) dorso-ventral position, and *θ*(t) growth angle. *θ *is defined as the deviation from longitudinal growth; positive values of *θ*(t) indicate a tendency to grow dorsally while negative values of *θ*(t) indicate a tendency to grow ventrally. In our first simple model just two parameters, *α *and *γ*, are defined specifically for each neuron type. The equations are:

*x*(*t *+ 1) = *x*(*t*) + Δcos(*θ(t)*),

*y*(*t *+ 1) = *y*(*t*) + Δsin(*θ(t)*),

*θ*(*t *+ 1) = (1 - *γ*)*θ*(*t*) + *ξ*,    *t *= 0,1,...,*n *- 1.

where *n *is the length of axon; ξ is a random variable uniformly distributed in the interval [-*α*, *α *] (*α *typically is about 2°-5°); and Δ is the 1 *μ*m distance grown in each time step. The parameter *γ *(0<*γ *<1) represents the tendency of an axon to turn towards an angle of 0 degrees – in other words, the tendency of the growth cone to orient towards longitudinal growth. We use aINs to illustrate our methods. Figure 6a shows aIN axons generated by the simple model for parameter values optimized using the procedure described below together with plots of the same number of real axons. It is clear that the simple model is able to generate the descending part of aIN axon growth (right part of the plot) but fails to fit the experimental data for ascending axons. This is because the descending aIN axons are mainly short with small turning angles while the ascending aIN axons are longer with larger turning angles. When all neuron types were considered we found that if model axons had appropriate tortuosities, then their dorso-ventral distributions were too broad and they often ran into the edges of the spinal cord.

The partial failure of the simple model suggested that, in life, some factors guide axons towards a longitudinal growth path and away from the edges of the cord. We therefore examined the turning angles of real axons (between points 0.05 mm apart) and found that they depended strongly on their current angle of growth and weakly on their dorso-ventral position. This is illustrated for aINs in Figure 6b,c where both scatter plots show negative correlations made clear by fitting the points by linear regression. For all measured neuron types the slope of the regression lines for axon turning angles were negatively dependent on current axon growth angle (-0.71 to -1.25) and dorso-ventral position (-0.08 to -0.53) (Table 3). This remarkable finding means, firstly, that the more an axon deviates from longitudinal growth the more it will turn back; secondly, the dependence of axon growth angle on dorso-ventral position means that for aIN axons, the upper and lower boundaries of the cord are repulsive.

**Table 3:** Dependence of axon turning angles on current growth angle and dorso-ventral position

	**Slope of turning angle versus**			
	
**Axons**	**Current angle**	*P*-value	**d-v position**	*P*-value
**RB**				
Descending	-1.15	0	-0.53	0
Ascending	-1.09	0	-0.57	0
**dlc**				
Descending	-1	0	-0.16	**0.2**
Ascending	-0.87	0	-0.15	0.007
**aIN**				
Descending	-0.95	0	-0.17	0.008
Ascending	-0.91	0	-0.08	**0.06**
**cIN**				
Descending	-0.71	0	-0.31	0.003
Ascending	-0.89	0	-0.12	0.031
**dIN**				
Descending	-1.15	0	-0.21	0.001
**mn**				
Descending	-1.25	0	-0.29	**0.13**

*P *= 0 means <0.0005. d-v, dorso-ventral. Non-sugnificant P values in bold.

In life many possible factors could influence axons to direct them away from edges (for example, physical barriers to growth cone extension, dorso-ventral gradients of repellent signals [38]) and guide them to a more longitudinal growth (for example, fasciculation with other longitudinal axons, longitudinal gradients of attractive or repellent signals [13]). We aimed to encapsulate the essence of such diverse mechanisms by introducing a new feature into our model: y¯
 MathType@MTEF@5@5@+=feaafiart1ev1aaatCvAUfKttLearuWrP9MDH5MBPbIqV92AaeXatLxBI9gBaebbnrfifHhDYfgasaacH8akY=wiFfYdH8Gipec8Eeeu0xXdbba9frFj0=OqFfea0dXdd9vqai=hGuQ8kuc9pgc9s8qqaq=dirpe0xb9q8qiLsFr0=vr0=vr0dc8meaabaqaciaacaGaaeqabaqabeGadaaakeaacuWG5bqEgaqeaaaa@2E3F@ represents the dorso-ventral position of an attractor to which axon trajectories are drawn with a strength of *μ*. Equations 1 and 2 are the same as above, but we replace equation 3 with:

*θ*(*t *+ 1) = (1 - *γ*)*θ*(*t*) - *μ*(*y*(*t*) - y¯
 MathType@MTEF@5@5@+=feaafiart1ev1aaatCvAUfKttLearuWrP9MDH5MBPbIqV92AaeXatLxBI9gBaebbnrfifHhDYfgasaacH8akY=wiFfYdH8Gipec8Eeeu0xXdbba9frFj0=OqFfea0dXdd9vqai=hGuQ8kuc9pgc9s8qqaq=dirpe0xb9q8qiLsFr0=vr0=vr0dc8meaabaqaciaacaGaaeqabaqabeGadaaakeaacuWG5bqEgaqeaaaa@2E3F@) + *ξ*,    *t *= 0,1,...,*n *- 1.

0 <*γ *< 1; 0 <y¯
 MathType@MTEF@5@5@+=feaafiart1ev1aaatCvAUfKttLearuWrP9MDH5MBPbIqV92AaeXatLxBI9gBaebbnrfifHhDYfgasaacH8akY=wiFfYdH8Gipec8Eeeu0xXdbba9frFj0=OqFfea0dXdd9vqai=hGuQ8kuc9pgc9s8qqaq=dirpe0xb9q8qiLsFr0=vr0=vr0dc8meaabaqaciaacaGaaeqabaqabeGadaaakeaacuWG5bqEgaqeaaaa@2E3F@ < 1

This model contains four parameters and to specify their values we used the following optimisation procedure. For each neuron type we first pick random values of the parameters and use these to generate 70 axons. After measuring the generated axons we calculate the tortuosities and the dorso-ventral distribution of axon positions just as for the experimental data. We then consider a cost function that includes the squared differences between experimental and generated axon distributions in 10% dorso-ventral bins and the squared difference between tortuosities. Using the optimisation process (see Methods), we find parameter values that minimise the cost function and give the closest match for each type of neuron. We repeat the same procedure to get optimal parameter values for each type of neuron separately for ascending and descending axons where both exist.

The revised model was able to generate axon growth patterns very similar to those in the spinal cord (for example aINs; Figure 6d). In many cases, the optimisation procedure was able to reach very small values of the cost function; for the few cases where it did not, the generated axons were still very similar to real ones. In addition, the modelled axon growth angles showed the same dependence on current angle and dorso-ventral position as the measured axons. Just as in the real axons, scatter plots and linear regressions showed negative slopes (Figure 6e,f; Table 3).

The second revised model of axon growth establishes that axon growth paths and distributions can be generated by very simple rules based only on the initial position and growth angle of the axons. Since these modelled axon distributions closely match those measured for real axons, it follows that their contact probabilities with dendrites will also be similar and we have confirmed this.

### Can simple connection rules specify functional spinal networks?

The results from recordings, anatomy and modelling of axon growth together suggest that early spinal networks may be able to develop using very simple rules. Since different types of spinal neurons have characteristic dorso-ventral positions for their cell-bodies, dendrites and axons, axons may not show any selectivity but simply synapse with a proportion of the dendrites that they contact. We therefore wanted to test if simple stochastic rules of connectivity could lead to functioning networks able to generate patterns of motor output suitable to produce swimming. Recent experiments in the immobilised hatchling *Xenopus *tadpole have shown that a very small part of the spinal cord and hindbrain, only 0.3 mm long, can generate long-lasting, alternating, swimming-like activity after a 1 ms current pulse stimulus [27]. This minimal preparation is not as reliable as more intact preparations and, as well as swimming, can also produce long-lasting rhythmic motor output that is synchronous on the left and right sides (SR Soffe, unpublished observations). Such synchronous activity has been seen in more intact tadpole preparations [39] and is a stable state in many simple reciprocal inhibitory network models [40,41]. Our aim was to use stochastic rules to build models of the minimal 0.3 mm long region of the tadpole nervous system to see whether they could produce similar rhythmic outputs, particularly swimming.

**Figure 7:**
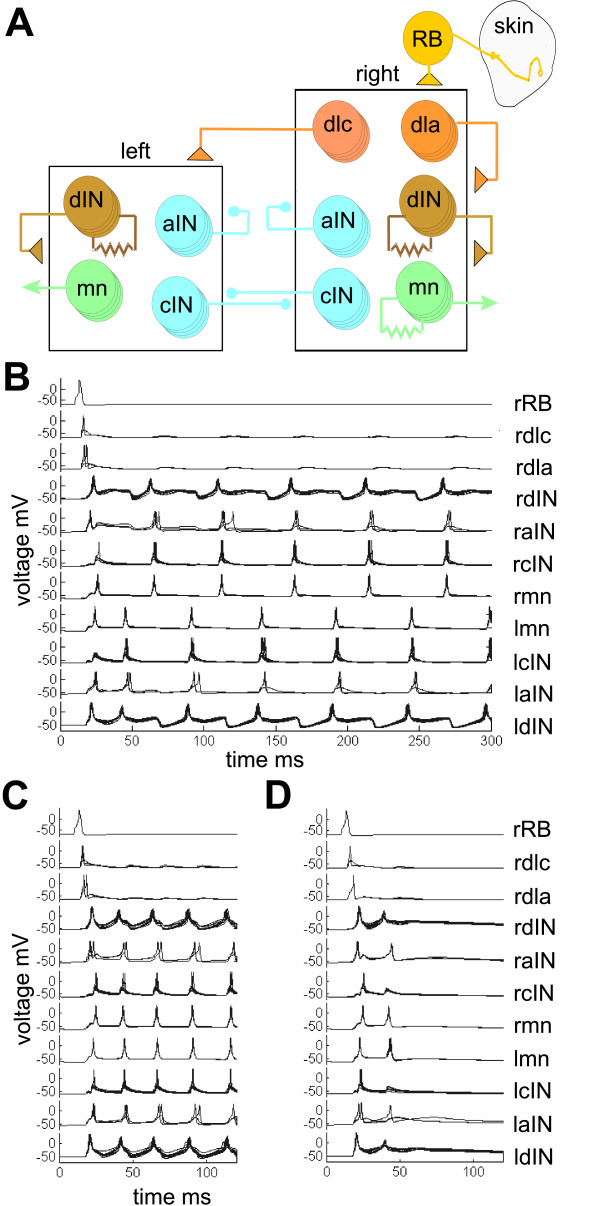
Model networks with probabilistic connectivity. **(a) **The network has a single sensory RB neuron exciting neurons in the right half-centre, which also has sensory pathway dlc and dla interneurons. There are ten of each neuron type in each half-centre. The broad pattern of connections is shown by the axons from groups of neurons onto the half-centres (triangles are excitatory and circles are inhibitory synapses). The actual synaptic connections are determined probabilistically for each neuron. Resistor symbols show electrically coupled neuron groups. **(b-d) **Examples of activity of selected neurons in response to a single stimulus to the sensory RB neuron for networks with connection probabilities based on experiments: (b) sustained swimming; (c) synchronous activity on each side; and (d) no long-lasting response.

We used model neurons with just a single compartment. Specific models were used for each type of spinal neuron with membrane properties and firing characteristics based on measurements from whole-cell patch recordings [42]. For simplicity we used a single RB neuron that excited all sensory pathway dlc and dla INs on the right side. Apart from RB neurons, there were ten of each type of neuron and the broad network connections are summarised in Figure 7a. The network has left and right sides that inhibit each other reciprocally. We have shown previously in a simple model of the rhythm generating part of the tadpole spinal network (CPG), that activating left and right sides with a delay leads to alternating swimming, but when the delay is too short, synchronous activity is produced from both sides [43]. The present network model could also produce swimming or synchronous activity, probably again dependent on the exact timing of activity in the sensory pathway. In the present model, synaptic connections were probabilistic and to imitate more realistic numbers of neurons (30 of each type) each model neuron had 3 chances to make a contact. Synaptic strengths could, therefore, be 0 or 1 to 3 times the single synapse strength (see Methods). Synaptic conductances and the ratios of NMDAR to AMPAR in glutamate synapses were specific for each type of post-synaptic neuron so that synapse properties matched those found in physiology (see Table in final Network modelling section of Additional file 1).

In the first test, 50 different networks were made where the synapse probabilities between different types of neuron were determined by those found experimentally (synapse probabilities from Table 2). The response of each network to a single stimulus to the sensory RB was then tested once. We found that 80% of these networks generated sustained activity: in 60% this was like swimming where left and right mns fired single, alternating action potentials on each cycle and the frequency was in the range of 10–30 Hz (Figure 7b); and 20% of networks produced synchronous output on each side at twice the swim frequency (Figure 7c). The remaining 20% failed to give any long lasting response to the stimulus (Figure 7d). Once initiated, swimming and synchronous activity continued indefinitely as there is no synaptic plasticity in these networks. In another 50 networks all synaptic connections were given the same contact probability, which was the average contact probability of the whole network. Swimming was never produced by these networks, even in networks where the high contact probabilities in the sensory pathway (connections from RB to dlc and dla) were restored.

We then investigated whether the particular synapse probabilities and strengths used in our first test of the model were critical to its success. Changing individual connections, especially in the sensory activation pathway, could reduce the percentage giving prolonged activity. For example, when the dlc to cIN synapse probability was reduced from 0.43 to 0.33, only 54% of networks produced prolonged activity (42% swim, 12% synchrony, n = 50). To see whether the broader pattern of synapse probabilities were important, we rounded-up all probabilities among aINs, cINs and dINs to 0.33 and found that 64% produced prolonged activity (26% swim, 38% synchrony, n = 50). These results show there is some flexibility in the values for synapse probabilities that can allow these stochastic networks to generate prolonged activity. The relatively high failure rates suggest that the sensory activation pathway is not nearly as secure in the model as in the whole animal. The reasons for this are not clear but the model network, although based on current evidence, is still unlikely to be a complete representation of the real networks.

We conclude that simple networks, where synaptic connectivity is determined by the broad dorso-ventral location pattern or geography of axons and dendrites, can generate organised swimming behaviour.

## Discussion

The hatchling frog tadpole can be used to study the specificity of connections between different types of neurons because recent technical advances have made it routine to record from pairs of neurons in the spinal cord. This has allowed the networks of spinal and caudal hindbrain neurons controlling swimming to be defined in considerable detail [23,28,25,31,27]. It would be rash to claim that all the neurons involved have been found; on the other hand, the evidence suggests that we have now defined all the major elements in the spinal network controlling swimming. Our knowledge of the neurons and connections in this example of a functioning vertebrate circuit producing meaningful behaviour provides a unique opportunity to ask questions about how such a circuit develops. We have therefore examined synaptic connections between seven different types of neuron. This allowed us to define the synaptic contact probabilities between these different neurons (Table 1). When considered in a functional context, most connections seemed very reasonable but, to our surprise, we found evidence for almost all possible connections. These observations do not rule out specific recognition processes acting during the formation of synaptic connections. However, they raise the possibility that simpler processes that lead to some 'mistakes' still provide connections with sufficient specificity to produce a properly functional circuit.

The simpler hypothesis that we have examined is that axons can recognise and make synapses with any dendrites that they contact, so the connections formed will depend primarily on the distribution of axons and dendrites. If this is correct, then synapse formation will occur where axons and dendrites lie in the same dorso-ventral regions of the spinal cord. Given the small scale of the tadpole spinal cord, which is only about 100 *μ*m in diameter, we have considered axons and dendrites to be within contact range if they simply lie within the same 10% dorsoventral position bins: approximately 10 *μ*m in the <10 *μ*m thick marginal zone of axons and dendrites. Clearly, more complex approaches are necessary in larger scale structures like the cerebral cortex [44,45]. On this basis, we therefore determined the anatomical contact probabilities of the axons and dendrites of different neuron types and compared these to the synapse probabilities determined directly by electrical recordings. The significant correlation between the two sets of probabilities suggested that synapses form in nearly 50% of cases when an axon passes through a dendritic field.

We conclude that axons make synapses with the dendrites they chance to contact rather than making synapses preferentially by recognising specific chemical markers on particular postsynaptic targets. Specific examples illustrate this. Skin sensory RB neurons drive a strong crossed excitatory reflex by first exciting sensory pathway excitatory dlc interneurons with commissural axons [30]. We might predict that connections from RB neurons to reciprocal inhibitory cINs would, therefore, be inappropriate and cell recognition factors might ensure they failed to form. Yet we find that these connections do form, and at a probability predicted simply by the overlap of RB axons and cIN dendrites. On the other hand, the excitatory connections between RB neurons and dlc interneurons occur with an unusually high probability [30]. Again, this is appropriate to drive a strong contralateral reflex and we might expect that this high contact probability would result from cell recognition. Yet the high probability can be explained by the distribution of dlc dendrites: unlike most spinal neurons, where dendritic fields are very narrow longitudinally, those of dlcs are unusual in being extended along the length of the cord [46]. This gives them repeated chances to make contact with RB axons. We suggest, therefore, that contacts are determined by the geography of the spinal cord, primarily by the dorso-ventral distributions of axons and dendrites.

If the dorso-ventral distribution of axons and dendrites is an important determinant of spinal network connectivity, then what are the factors that control these distributions? Fortunately, this is a very active area of research. Different dorso-ventral distributions of axons and dendrites originate with the specification of soma positions. In the chick and mouse, a large body of work is defining the transcriptional networks that regulate the formation of an ordered dorso-ventral series of longitudinal neuron columns identified by the transcription factors that they express [47,7,8,49]. Fundamentally, around 12 neuron types are arranged in a consistent sequence of columns from dorsal to ventral: sensory, sensory related interneurons, motor related interneurons and mns. The same basic plan is seen in the tadpole spinal cord (Figure 1c,d). Once formed into these columns, neurons are polarized [9] and grow processes in very distinct orientations. In frogs, most grow axons ventrally (with the obvious exception of the sensory RB neurons that grow longitudinally [46]). Growth cones immediately come under the influence of attractive and repulsive chemical gradients that control their direction of growth, for example, whether they turn or grow straight across the ventral surface to the opposite side before turning [1,50,4,38,52]. In the tadpole all axons eventually grow in a longitudinal direction, starting in a characteristic dorso-ventral region for each neuron type. Meanwhile, dendrites grow from the soma or initial segment of the axon and, like the axons, come to lie in dorso-ventral positions characteristic for each neuron type. In contrast to extensive studies on dendrite development in brain neurons [53], there is little work on the mechanisms determining their growth in spinal neurons. Evidence from zebrafish shows that dendrites play an active role in extending very short distances (approximately 10 *μ*m) towards longitudinal axons to form en-passant synapses [54].

To test the plausibility of the proposal that synapse formation between different neuron types in the tadpole spinal cord depends on the dorso-ventral distribution of axons and dendrites, we have used two types of modelling. Since axons grow long distances along the cord, they could wander to reach all dorso-ventral positions unless their growth is regulated. We therefore asked what kinds of growth rules were needed to reproduce the patterns of axon distribution found for different neuron types. A simple tendency to turn towards longitudinal growth could not match the real axon distributions or their active turning responses, which change with growth angle. To match the real axons we needed to add active turning towards an attractor line located at different dorso-ventral positions for each neuron type. This attractor models the complex effects of interacting attractive and repellent dorso-ventral chemical gradients that have been proposed to act on axonal growth cones in the spinal cord [4,1]. With the attractor, our simple model could reproduce real axon distributions and, by doing this, could reproduce the synaptic contact probabilities determined anatomically. (These assume that dendrite distribution is static and passive, which is almost certainly not the case.)

In our second modelling test we asked whether a functional spinal network capable of generating swimming activity could be generated simply on the basis of the synaptic contact probabilities established by our physiological and anatomical data on the different spinal neuron types. It is important to remember that in our network model, the different neuron types are not all alike but each type has their own very particular and characteristic properties [42]. Using these neurons, we show that crude probabilistic contact rules do produce networks that will generate swimming activity while networks where all neurons have the same connection patterns fail. Taken together, the modelling supports the proposal that functional circuits could be produced using simple rules. For example: excitatory dIN axons should grow tailwards, mainly in the ventral 50% of the cord and synapse with 40% to 50% of any dendrites passed; reciprocal inhibitory cINs should cross the cord ventrally, branch on the other side, grow mainly in the ventral 50% of the cord and synapse with 40% to 50% of any dendrites passed. It is important to emphasise that we are not suggesting that chemical recognition does not exist; axons need to recognise dendrites. We are suggesting that detailed cell-to-cell recognition may not be necessary to establish which connections are made in the first, functional pioneer circuits.

## Conclusion

In the core, axial parts of the vertebrate nervous system, like the spinal cord and brainstem, neurons, dendrites and longitudinal axons are laid out in a dorso-ventrally ordered array on each side of the body. At early stages in development a major factor influencing primary synapse formation in such regions may be the physical proximity or separation of axons and dendrites. If axons can recognise and contact dendrites, then synapses may form. So, in the frog tadpole spinal cord, dorsally located sensory axons mainly excite the dorsal dendrites emerging from the cell bodies of dorsal sensory pathway neurons (dlcs) but the very ventral central axons of mns will virtually never contact these dendrites, so synapses will not be made. At this early, primary stage of development neurons may need only to be able to distinguish neuronal dendrites from axons and non-neuronal processes. Detailed cellular recognition and other more subtle processes to specify correct connections may, therefore, not be necessary for the formation of primary functional networks during spinal cord development. This lack of specificity could be tested if different types of spinal neurons could be marked and would form synapses in culture. Such recognition processes surely play important roles at later stages of development as connection patterns are refined [55,19].

## Methods

### Physiology: whole-cell patch recording

Details of the recording methods have been given recently [28]. Briefly, *Xenopus *tadpoles at stage 37/38 (Figure 1a) were anaesthetised with 0.1% MS-222 (3-aminobenzoic acid ester; Sigma, Poole, UK), immobilized in 10 *μ*M α-bungarotoxin saline, then pinned in a bath of saline (concentrations in mM: NaCl 115, KCl 3, CaCl_2 _3, NaHCO_3 _2.4, HEPES 10, adjusted with 5 M NaOH to pH 7.4). Saline with 0 mM Mg^2+ ^was used so NMDAR mediated components could be seen. Skin and muscles over the right side of the spinal cord were removed and a mid-dorsal cut made along the spinal cord to open the neurocoel. Small cuts were made in the wall of the neurocoel on the left side to expose more ventral neurons. The tadpole was then re-pinned in a small 2 ml recording chamber with saline flow of about 2 ml per minute. Exposed neuronal cell bodies were seen using a ×40 water immersion lens with bright field illumination on an upright Nikon E600FN microscope. Antagonists were applied close to the recorded neuron soma using gentle pressure to solution in a pipette with a tip diameter of 10–20 *μ*m or dropped into a 200 *μ*l well upstream of the recording chamber. Drugs used were NBQX (2,3-dihydroxy-6-nitro-7-sulfamoylbenzo- [*f*]quinoxaline- [*f*]quinoxaline, Tocris), D-AP5 (D-(-)-2-amino-5-phosphonopentanoicacid, Tocris), bicuculline, strychnine, tetrodotoxin, d-tubocurarine and mecamylamine (Sigma) and DHβE (dihydro-β-erythroidine; Research Biochemicals International, Natick, MA, USA).

Patch pipettes were filled with 0.1% neurobiotin and 0.1% Alexa Fluor 488 (Invitrogen, Eugene, OR, USA) in intracellular solution (concentrations in mM: K-gluconate 100, MgCl_2 _2, EGTA 10, HEPES 10, Na_2_ATP 3, NaGTP 0.5 adjusted to pH 7.3 with KOH) and had resistances around 10 MΩ. Junction potentials were corrected before making recordings. Signals were recorded with an Axoclamp 2B in conventional bridge or continuous single electrode voltage clamp mode, acquired with Signal software through a CED 1401 Plus interface with a sampling rate of 10 kHz (Cambridge Electronic Design, Cambridge, UK). Offline analyses were made with Minitab (Minitab Ltd, Coventry, UK) and Microsoft Excel. All values are given as mean ± standard error of the mean. Experiments complied with UK Home Office regulations and received local ethical approval.

### Anatomy

Neuron anatomy in *Xenopus *tadpoles at stage 37/38 was revealed by two methods. In the first method, mns were backfilled by applying fluorescein dextran to their axons in the swimming trunk muscles. After 10 minutes, muscle was removed to allow access to the side of the spinal cord and living mns observed and photographed on a Bio-Rad 500 confocal microscope with a ×40 water immersion lens [56]. In the second method, all other neurons were filled with neurobiotin through recording microelectrodes [57]. After fixing and processing, the CNS was exposed and specimens mounted on their sides between coverslips for observation, tracing with a drawing tube, or photography at ×500 on a bright field microscope [28].

Tracings of the soma, dendrites and full axonal projections were made to a scale of 0.1 mm = 50 mm. We used these scale drawings of neurons located 1 to 3 mm from the midbrain to record the dorso-ventral positions of soma, dendrites and axons for each type of neuron. The distances measured were: from the soma to the midbrain/hindbrain border; from the dorsal to ventral edge of the cord at the level of the soma; from the dorsal edge of the soma to the ventral surface of the cord; from the ventral edge of the cord to the most dorsal and most ventral dendrite. On each side of the soma, the distance of the axon from the ventral surface of the spinal cord was measured every 0.05 mm. All measurements on fixed specimens were multiplied by 1.28 to compensate for shrinkage during dehydration [24].

### Modelling axon growth

The traditional approach to modelling axon growth is based on the growth cone following molecular gradients [58]. Instead, we build a simple computational model reflecting several key attraction and repulsion processes guiding axon growth. The model includes four parameters (see equations 1, 2 and 4) that should be chosen to provide a maximum similarity between experimentally measured axons and model generated axons. Fitting the model to experimental measurements is least squares based and the similarity measure (or cost function to be minimised) contains two terms: the first term compares the distribution of model axon dorso-ventral coordinates with the distribution of experimental coordinates; the second term takes into account the extent to which the axon path is circuitous rather than direct by comparing the model and experimental tortuosities. A detailed description of the cost function to be minimized in order to find optimal parameter values is given in Additional file 1.

We run the optimization procedure for the ascending and descending axons of each neuron type. This process gives four optimal parameter values that we then use to generate biologically realistic axons with distributions of dorso-ventral coordinates and tortuosities similar to measured axon characteristics for each neuron type. We test the optimal parameter values to define the reliability of the optimization. Testing reliability is an important part of the modelling procedure because the model includes a random component in equation 4. If, for example, we generate two sets of axons using the same optimal parameters, we would like to be sure that these sets are similar. We also ensure that the properties of generated axons are not affected by 5% to 10% changes in model parameters. Optimal parameter values for each cell type, a detailed description of the testing procedure and results of testing are given in the Axon modelling section of Additional file 1.

### Modelling spinal networks

Ten neurons of each type are modelled on each side [42], but at the level of connectivity each neuron represents three neurons that fire synchronously. Thus, effectively 30 neurons of each type are modelled on each side. Neurons with an ipsilateral axon connect only to ipsilateral neurons, while those with commissural axons connect only to neurons on the opposite side. After these restrictions, connections are made purely on probabilistic rules, using the contact probabilities from Table 2 ('synapse' probabilities). Since each neuron represents three synchronously firing neurons, each neuron has three chances to connect to any other neuron, where at every single attempt, there is the same probability of actual contact being established.

The connection strengths of individual synapses are primarily based on the strengths used in [42], where these strengths resulted in realistic overall conductances during swimming. These strengths had to be reduced to account for the probabilities of contact within the network, and the multiplicity of synaptic contacts between two types of neurons. The actual maximum conductances during swimming can be computed only at run-time, and vary with the connection pattern of the network, according to the probabilities of contact (the parameters used are given in a Table in the Network Modelling section of Additional file 1). Resistive electrical synapses are established between dINs and between mns with a strength of 0.3, meaning that a change of 10 mV in one neuron will cause a change of 3 mV in the other one.

## Competing interests

The author(s) declare that they have no competing interests.

## Authors' contributions

W-CL carried out the electrophysiology, contributed to the anatomical study and analysis of recordings. TC and RB carried out the analysis of the axon pathways and their modelling. BS carried out the network modelling. SRS participated in the design of the study and the analysis of physiology and anatomical results. AR conceived of the study, participated in its design and coordination, and was lead writer of the manuscript. All authors contributed to writing and figure preparation and have read and approved the final manuscript.

## Supplementary Material

Additional file 1 Additional material referred to in the text, in particular concerning the axon pathway modelling. Click here for file
