# Supplementary material for: Axon and dendrite geography predict the specificity of synaptic connections in a functioning spinal cord network
Source: Neural Dev. 2007 Sep 10;2:17. doi: 10.1186/1749-8104-2-17 (PMC2071915; doi:10.1186/1749-8104-2-17)
Supplement: Additional file 1 — Additional material referred to in the text, in particular concerning the axon pathway modelling. [file 1749-8104-2-17-S1.pdf]

Li et al., Supplementary Material

1) Anatomy

Table showing distribution of axons and dendrites of different neuron types. The cord is divided dorso-ventrally into 10% bins. The table then shows the probability of axons and dendrites of neurons of each type occupying each of these bands.

**axons**	RB	dlc	aIN	cIN	dIN	mn
position %						
100	0.10	0.00	0.00	0.00	0.00	0.00
90	0.23	0.00	0.03	0.00	0.00	0.00
80	0.29	0.00	0.08	0.00	0.00	0.00
70	0.27	0.03	0.15	0.03	0.01	0.00
60	0.07	0.13	0.18	0.11	0.14	0.00
50	0.00	0.12	0.21	0.17	0.15	0.00
40	0.00	0.26	0.23	0.22	0.43	0.00
30	0.00	0.26	0.06	0.22	0.18	0.15
20	0.00	0.17	0.04	0.17	0.09	0.72
10	0.00	0.04	0.01	0.08	0.01	0.13
n =	*10*	*10*	*10*	*9*	*10*	*11*
**dendrites**		dlc	aIN	cIN	dIN	mn
position %						
100		0.10	0.00	0.00	0.00	0.00
90		0.80	0.20	0.00	0.10	0.00
80		0.90	0.30	0.00	0.20	0.00
70		0.60	0.40	0.22	0.60	0.09
60		0.40	0.70	0.67	0.70	0.27
50		0.10	0.90	0.78	0.70	0.64
40		0.00	1.00	0.89	0.80	0.91
30		0.00	0.90	0.56	0.40	1.00
20		0.00	0.90	0.44	0.20	1.00
10		0.00	0.80	0.00	0.10	0.82
n =		*14*	*10*	*9*	*10*	*11*

2) Modelling axon growth

A. Introduction

The traditional approach to modelling axon growth is based on the idea that the growth cone follows different molecular gradients (Goodhill et al., 2004; Krottje and van Ooyen, 2007). Here we do not consider the details of growth cone navigation in steep and shallow chemical gradients; instead we build a simple computational model reflecting several key attraction and repulsion processes guiding the axon development. As specified in the main text, the model is described by a dynamical system with a random variable (see equations (1), (2) and (4)) and includes four parameters.

In this Appendix we will describe the optimization procedure which allows us to find a set of optimal parameter values. As usual optimality means minimising a cost

function. Thus, starting from discussion of parameters we will define the cost function which measures the similarity between experimental axon measurements and model generated axons, describe the optimization procedure which minimises the cost function and provides the best similarity of experimental and generated model axons, report the optimal parameter values for each neuron type, and demonstrate that optimal parameter values allow us to generate model axons which are similar enough to the biological axons measured in experiments (testing procedure).

B. Model explanation

Experimental measurements have been made for different tadpoles with different spinal cord heights which are about 100 μm. To reflect that in the model, we consider axon growth in one side of the spinal cord represented as a rectangle with some height *H* randomly chosen in the range of admissible biological height values (about 100 μm) and length *W*= 1000 μm (this length is the same for all model simulations, therefore, when an experimental axon is longer than *W* , this axon was cut to have length exactly *W*= 1000 μm). Thus, we consider the rectangle *H* x *W* and growing axons are allocated inside of this field. To start an axon simulation we need to choose the initial position of the axon and initial angle, after that a process of axon generation is governed by model equations (1), (2), (4) described in the main text.

Developing the model equations, we implicitly assume that chemical gradients experienced by the growth cone are exponential, which for a single gradient would produce a constant rate of turning independent of the location within the gradient (but not independent of the current growth angle). The dependence of axon growth angle on dorso-ventral position (note, that dorso-ventral axis corresponds to vertical axis (height) of the rectangle and longitudinal location is considered to lie along the horizontal axis (length) of the rectangle) that we observe is assumed to be the consequence of interaction between at least two gradient-following processes: the noise in the axon growth angle and the tendency to grow towards some particular location. The noise component describes a random deviation of the current angle from deterministic component (see equation (4)). Thus, the noise component is a random variable uniformly distributed in the interval $(-\alpha, \alpha)$, where parameter α defines the boundary for the angle deviation (Fig. S1). Thus, the noise is modelled by a uniformly distributed random variable with mean equal to zero and variance equal to $\alpha^2/3$.

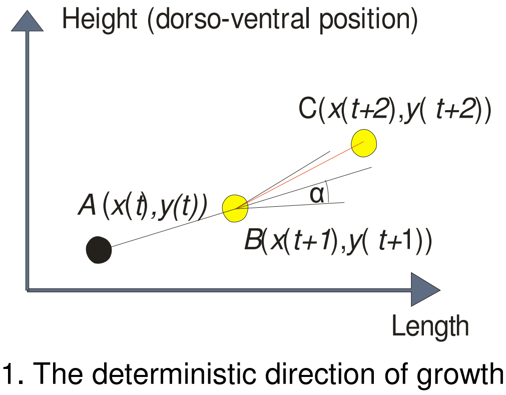

Fig. S1. The deterministic direction of growth is shown by black line connecting point A and the yellow dot. The angle specifies boundaries of random deviation. The red line shows the chosen direction of growth for the next time step.

The parameter γ represents the tendency of the axon to turn towards an angle of 0 degrees – in other words the tendency of the growth cone to orient towards

longitudinal growth. If γ is zero (see equation (3) in the main text) then the deterministic part of the growth angle is not changed at each time step and random deviation applies to this direction. When 0< γ<1, the deterministic component of the growth angle will decay to zero. This part of the model can be justified by experimental findings which show that this orienting process towards zero angle is dependent on the current deviation from longitudinal growth – the steeper the current growth angle, the stronger the tendency to straighten towards horizontal growth. The effect of parameter γcan also be interpreted as the consequence of a longitudinal gradient-following process, which would be expected to produce the same dependence on growth cone angle.

The parameter $\bar{y}$ represents the dorsoventral position of an attractor to which axon trajectories are drawn with a strength which can be described by parameter μ (see equation (4)). Thus, parameters μ *and* $\bar{y}$ characterise the interaction between two opposing gradient-following processes. The parameter $\bar{y}$ is the dorsoventral position at which these processes effectively cancel each other out. The parameter μ represents the strength of the net attraction towards $\bar{y}$. The effects of these parameters can be interpreted as a system with two repulsive gradients, one pushing from the ventral side to the dorsal direction (we know that there is some drive here at least with the commissural neurons) and one pushing from the dorsal side to the ventral direction. The relative sensitivity of the axon to these two gradients would determine the value of the parameter $\bar{y}$ and the absolute sensitivity of the growth cone to ligands would determine the value of the parameter μ.

C. Preparation for optimization

Our goal is to simulate an axon growth process which can generate axons that are similar to biological axons which are measured experimentally. Similarities are measured using a cost function with two components: 1) similarity of distributions of axon projections in the dorso-ventral dimension and 2) similarity of tortuosities. Thus, the optimization procedure looks for a set of values of the four model parameters which provide the minimum cost function. Below we formulate the optimization procedure in detail.

The available experimental data provide measurements of spinal cord axons for each neuron type in both descending and ascending directions when both are present. We will consider aIN ascending axons as an example to demonstrate how the optimization procedure works. Available experimental data provide measurements of 10 axons from different tadpoles. The longitudinal dimension in the model was always 1000 μm however axons can be shorter or longer than this full length. Experimental measurements of dorso-ventral axon position (in micrometers) were made every 50 μm. An example of a measured axon is shown in Fig. S2. The horizontal red line (y=94.7 μm) shows dorso-ventral height boundary of the spinal cord and the vertical red line shows the longitudinal boundary of considered spinal cord measurements.

The next step is the projection of axon measurements in the dorso-ventral direction and the calculation of the distribution of these projections. Because the dorso-ventral height of the rectangles varies for different axons extracted from tadpoles with different length of the spinal cord, we normalise axon coordinates before projecting them. Thus, we normalise both axes by dividing both vertical and horizontal axon coordinates by the dorso-ventral height of the spinal cord (in this example the height H=94.7 μm). Division of both coordinates allows us to keep the angle structure

unchanged. Of course, after this transformation a step along the horizontal axis will be different from 50 µm however the image representation will be the same.

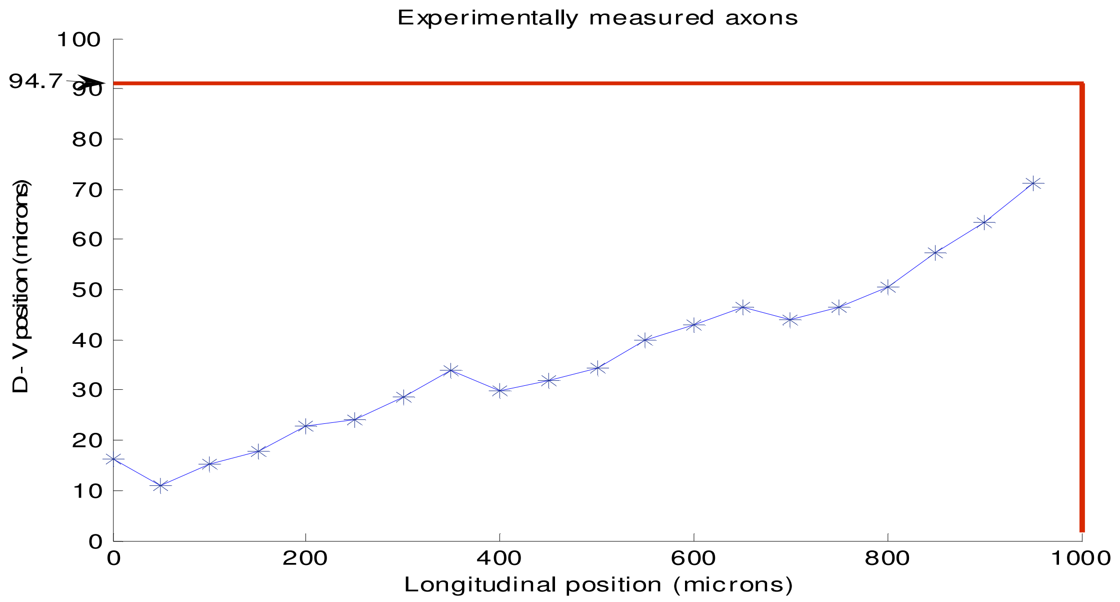

Fig. S2. An example of axon measurements

After normalization we project all aIN ascending axon data to the vertical axis and repeat this procedure for each axon. The total number of axon measurements is n_e $(n_e = 167)$ and these data represent the dorso-ventral distribution of axon measurements in the interval 0-100. We divide this interval into 10 bins, count the number of measurements in each bin, and normalize it by the total number m to get a probability of finding an axon measurement in the bin. The resulting distribution is shown in Fig. S3.

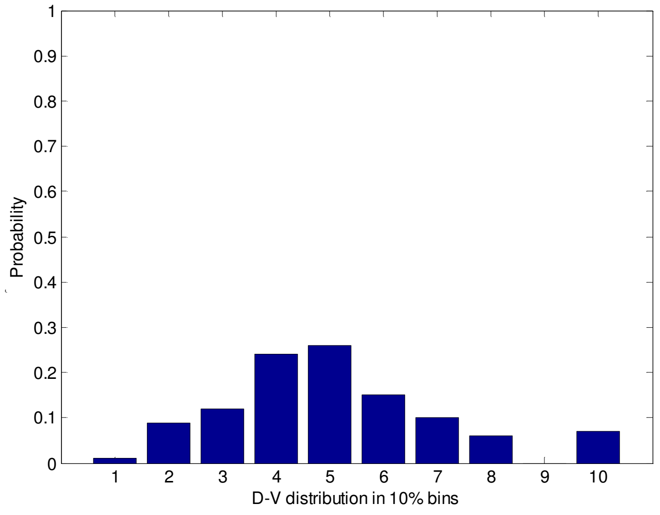

Fig. S3. An example of the distribution of measured D-V coordinates for aIN ascending axons.

Moreover, we would also like the cost function to take into account the extent to which the path of the axon is circuitous rather than direct. Results from multiple

model simulations suggest that tortuosity (total path length divided by straight line distance between start and end points) is an appropriate measure for this purpose. Thus, we calculate the tortuosity of each axon using the following formula:

$$T = \frac{\sum_{i=1}^{k} \sqrt{(x_i - x_{i-1})^2 + (y_i - y_{i-1})^2}}{\sqrt{(x_k - x_0)^2 + (y_k - y_0)^2}},$$

where (x_i, y_i), $i = 0,1,...,k$ are measured coordinates of the axon, and k is the number of measurements for the axon. After that we calculate the average tortuosity of experimental axons $\bar{T}^e$.

Now we would like to describe the process of axon generation. Suppose that values of the four parameters of the model are known, then we can start the process of axon generation described by equations (1), (2), and (4) in the main text. For that we need to choose initial values for variables of the dynamical system, i.e. coordinates of the starting point of the axon (x_0, y_0) and the initial growth angle θ_0. Also, we need to fix the length of the generated axon.

For generation of all axons we choose the same initial point in the horizontal axis: $x_0 = 0$. To choose the initial value of the vertical coordinate, we first calculate the sample distribution (10 bins for 0-100 interval) of normalised initial vertical coordinates of all experimentally measured axons and generate a random number ran according to this distribution, thus, $y_0 = ran$. After that, to choose the initial angle, we consider the bin of distribution where ran is and study initial angles $\eta_1,...,\eta_q$ of axons which have the vertical coordinate of their starting point in this bin. We generate uniformly distributed random angle η in the interval $(\min(\eta_1,...,\eta_n), \max(\eta_1,...,\eta_n))$ and the initial growth angle $\theta_0 = \eta$. Similarly, for the axon length we build the distribution of experimental axon length and generate the random number according to this distribution. Also, we use the same procedure to generate dorso-ventral height of the spinal cord: we build the distribution of experimentally measured dorso-ventral heights and generate the random number (*ran_height*) according to this distribution; thus, we have chosen the rectangle (*ran_height* x 1000) where all model generated axons will be allocated, i.e. we use the same rectangle to generate several axons and allocate them to the same rectangle.

After fixing all initial values and axon length we run the system of difference equations (1), (2), (4) in the main text and generate an axon. For axon generation we use step Δ = 1 μm. To get generated axon data similar to the experimental measurements we sample model axon coordinates every 50 μm and use these sampled data for the following procedures: projection of axons, building D-V distribution, calculation of tortuosity, etc. Fig. S4 shows an example of a generated model axon for the optimal parameter values fitted to aIN ascending experimental measurements. Green line shows generated model axon with 1 μm step, markers show measurements at 50 μm steps along the horizontal axis, the same sampling as in experiments. The lower panel of Fig. S4 shows the same generated axon in the “correct” scale where vertical and horizontal axes are proportional and angles are not distorted.

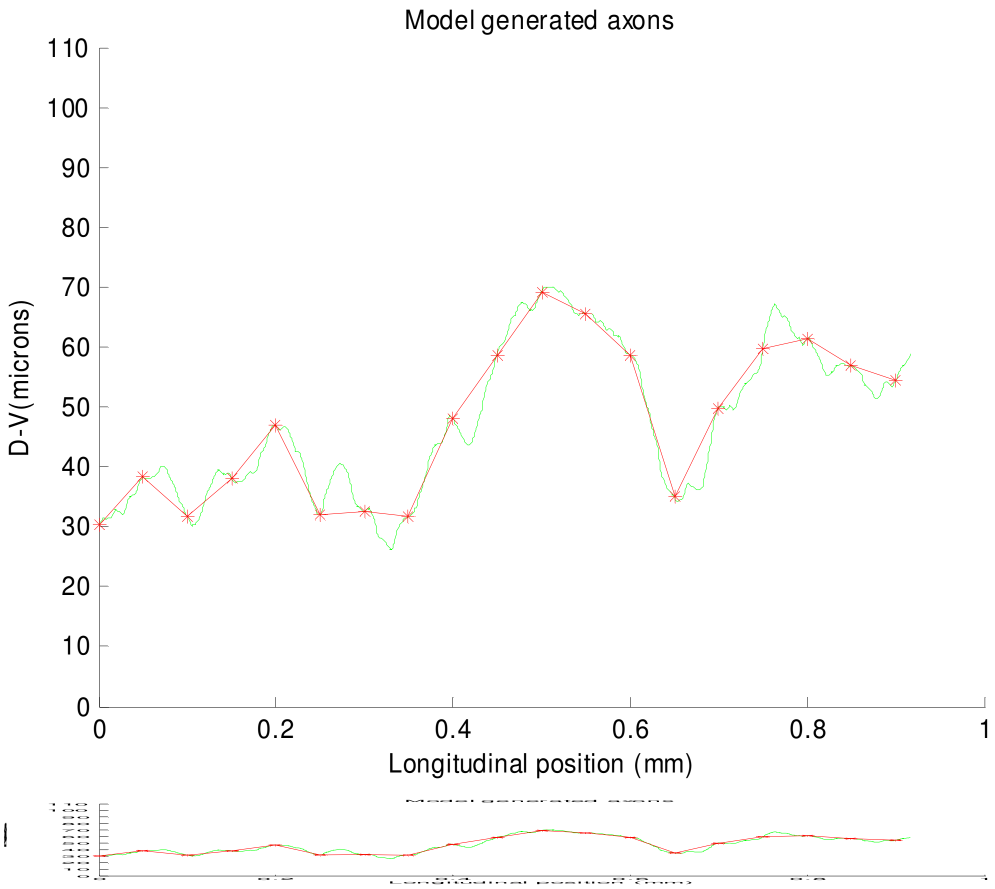

Fig. S4. Example of generated axon for optimal parameter values of aIN ascending neuron type. Green line shows generated axon and red line shows approximation with 50 μm sampling along the horizontal axis. Lower panel shows the same axon with proportional axes.

It is important to note that the procedure for choosing initial values and axon length involves generating random numbers. This means that repetition of the same procedure will result in the generation of a different axon with different initial values and a different length. Thus, we repeat this procedure *r* times (*r* =70), generate *r* axons allocated inside the same rectangle, and calculate the dorso-ventral distribution (10 bins for 0-100 interval) of all vertical coordinates of all generated axons. This distribution we denote by $(y_1^m,..., y_{10}^m)$, and the total number of coordinates used for calculation of the distribution is n_m (index *m* here means relates to 'model'). Also, for each model axon we calculate the tortuosity and find the average tortuosity of generated axons $\overline{T}^m$.

Now we can define the <u>cost function</u> which includes both similarity of distributions and similarity of tortuosities. To measure similarities of distributions we use a sum of squared differences between experimental and modelled distributions. To make a guess about possible values of such a sum, we would like to use normalization which is traditionally used in statistics for comparing distributions and representations of random variables with chi-square distribution. Thus the first term of the cost function is:

$$f_{chi} = \sum_{i=1}^{10} \frac{(y_i^e - y_i^m)^2}{y_i^e n_e + y_i^m n_m},$$

where y_i^e , y_i^m are projection counters for bin i for experimental and model results respectively, $i = 1,...,10$, n_e and n_m represent the numbers of experimental and model results respectively.

It is known in statistics that the upper boundary of 95% confidence interval for hypothesis testing about similarity of two distributions is 3.9. Thus, this value can serve as guidance for understanding the scale of cost function values and judging the quality of the optimization process.

The second term of the cost function is the squared difference between average experimental tortuosity $\overline{T}^e$ and average model tortuosity $\overline{T}^m$. The two terms of the cost function have very different scales and to balance them we consider a weight coefficient w which makes these terms consistent and with values in the same interval. Thus, the final expression for the cost function is:

$$f_{cost} = f_{chi} + w(\overline{T}^e - \overline{T}^m)^2 ,$$

where $w = 10^5$.

It is worth noting that the cost function includes a stochastic component, therefore, repeated calculation for the same parameter values will always result in different values of the cost function. Thus, gradient based methods are not appropriate for optimization because they usually require the cost function to be smooth which it is not in our case. We use the Nelder-Mead simplex method which can deal with cost functions that are non-smooth, even if they include a stochastic component.

D. Numerical algorithm for optimization: The Nelder-Mead simplex method

To solve the optimization problem and find parameter values which provide a minimum cost function, we use the MATLAB procedure “fminsearch” which is based on the Nelder-Mead simplex method (Lagarias et al., 1998).

Let us formulate the idea of the simplex method. For two variables the simplex is a triangle, and the method is a pattern search that compares function values at the three vertices of a triangle. The worst vertex, when the function value is largest, is rejected and replaced with a new vertex. A new triangle is formed and the search is continued. The process generates a sequence of triangles (for which the function values at the vertices get smaller and smaller). The size of the triangles is reduced and the coordinates of the minimum point are found. The algorithm is stated using the term simplex (a generalised triangle in *N* dimensions) and will find the minimum of the function of *N* variables. It is effective and computationally compact.

The description of the algorithm

Let $f(x, y)$ be the function that is to be minimised.

To start we are given 3 vertices of a triangle $(x_k, y_k),\ k = 1,2,3.$ The function values $z_k = f(x_k, y_k),\ z_1 \le z_2 \le z_3$.

Best vertex $B = (x_1, y_1)$.

Good vertex $G = (x_2, y_2)$.

Worst vertex $W = (x_3, y_3)$.

Mid-point of the good side:

$$M = (B+G)/2 = \left(\frac{x_1+x_2}{2}, \frac{y_1+y_2}{2}\right).$$

Reflection using point *M:*

$$R = M + (M - W) = 2M - W.$$

Expansion*:*

$$E = R + (R - M) = 2R - M$$

Contraction using point the point C

$$C_1 = (W+M)/2;\; C_2 = (M+R)/2;\; \text{if } f(C_1) < f(C_2) \text{ then } C = C_2 \text{ else } C = C_2.$$

Shrink toward B

$$S = (W+B)/2.$$

Logical decisions:

$$\text{If } f(R) < f(G) \text{ then case I}$$
$$\text{else case II}$$

Case I

$$\text{If } f(B) < f(R) \text{ then replace } W \text{ with } R$$
$$\text{else compute } E \text{ if } f(E) < f(B) \text{ replace } W \text{ with } E \text{ else replace } W \text{ with } R$$

Case II

$$\text{If } f(R) < f(W) \text{ then replace } W \text{ with } R.$$
$$\text{If } f(R) \geq f(W) \text{ then compute } C, \text{ if } f(C) < f(W) \text{ then replace } W \text{ with } C$$
$$\text{else compute } S, \text{ replace } W \text{ with } S, \text{ replace } G \text{ with } M$$

E. Results of optimization and testing the optimization quality

It is important to note that the result of the optimization procedure is a random variable. This means that if we have found a set of optimal parameter values and use them to calculate the cost function several times, we will get different cost function values, because the random number generator will start from different initial values resulting in generation of different axons. Thus, we would like to test the result of optimization studying the distribution of the cost function values generated for the set of optimal parameter values.

The optimization procedure was run for each cell type and separately for their descending and ascending axons. The best values of model parameters and quality of optimization are summarised in the Table 1. To characterise quantitatively the quality of optimization we define the measure Q in the following way. One trial of the testing procedure includes the generation of 300 axons for the optimal parameter values and calculation of the cost function. We repeat this procedure 100 times, generate 100 values of the cost function, and build a histogram which we call the testing histogram (examples are given in Section 6). We denote by Q a value of the cost function such that the interval (0,Q) corresponds to 90% of the area of the histogram. We consider the optimisation procedure to be: *very good* quality if Q is less than 4; *good quality* if 4<Q<8.5;and *poor quality* if Q>8.5. The last two columns

of Table 1 show the quantitative and qualitative characteristics of the optimization procedure for each cell type.

Table 1 Parameters for model axon generation and quality of optimization

	alpha	gamma	mu	ybar	quality	Q
aIN des	0.10370925019052	0.11917600682222	0.01181789936320	0.55117617500812	good	8
aiN as	0.23732586251142	0.08813752706822	0.02673982589197	0.69775906459744	good	8
cIN des	0.05375985714918	0.06152852069920	0.01391706049223	0.73589974835070	good	6
cIN as	0.05905177737630	0.08263326038516	0.01091760433793	0.71105411267945	good	4.5
dIN des	0.12185512189028	0.09565323676794	0.02108649340963	0.38062246228996	poor	21
RB des	0.11645497350869	0.04533838635712	0.05581374613877	0.69817607934022	poor	18
RB as	0.12235692069202	0.04323400746809	0.04999578629642	0.79172680584209	good	6
dlc des	0.14191302051465	0.09199435732217	0.04112664456976	0.41155539040449	good	6
dlc as	0.11357299174507	0.11448935909590	0.01791283546036	0.65003923169086	poor	17
MN	0.10475413875442	0.41730415071674	0.02818952435167	0.17638544195660	very good	1.5

Sensitivity of optimal parameter values to parameter variation

Following the optimization process, we tested the sensitivity of the optimal parameter values to small variations. We consider 3 levels of variation: 5%, 10% and 20% (accordingly, the fractions of variation are Fr=0.05, Fr=0.1; Fr=0.2) and for each level, (e.g. 5%), we consider three cases for each of the four parameters: 1) the parameter value decreases by 5% (*index*= -1); 2) the parameter value is not disturbed (*index*=0); 3) the parameter value increases by 5% (*index*=+1). The following formulas show how we calculate variations of the optimal parameter values for testing:

$$vpar_k = opar_k + index_k * Fr * opar_k,\ k = 1,2,3,4;$$
$$index_k \in \{-1,0,+1\};\ \ Fr \in \{0.05,\ 0.1,\ 0.2\},$$

here $vpar_1,\ opar_1$ correspond to perturbed and optimal values of the model parameter α respectively; $vpar_2,\ opar_2$ correspond to perturbed and optimal values of the model parameter γ respectively; $vpar_3,\ opar_3$ correspond to perturbed and optimal values of the model parameter μ respectively; $vpar_4,\ opar_4$ correspond to perturbed and optimal values of the model parameter $\bar{y}$ respectively; the value of $index_k$ can be taken from the designated set $\{-1,0,+1\}$ and *Fr* can also take any of three possible values $\{0.05,\ 0.1,\ 0.2\}$ indicating different degrees of variation. Table 2 shows the performance of the model for different degrees of parameter variation in terms of the measure Q (described above), for the case of cIN ascending axons. The first column of the table shows the sequential number of the test case (1-81), the following 4 columns show the values of $index$ for each parameter. For example, the first row shows that the values of all four parameters have been decreased, the second row shows the test case when the values of $\alpha,\ \gamma,\ \mu$ have been decreased but the value of $\bar{y}$ has not been changed, etc. The last three columns show the quality of optimization Q for different levels of variation (5%,10%,20%).

This table indicates that for 75% of these cases at the 5% variation level, the value of Q remains below 8.5, which is our criterion for good quality results. At the 10% variation level, 42% of cases produce good quality results, and even at the 20% variation level, about 21% of cases give good quality results. In this particular case of the cIN ascending axons considered here, the poorest results occur when $\bar{y}$ is reduced (made more ventral), especially when μ is decreased at the same time, as in these cases the axons do not reach a sufficiently dorsal position to adequately match the experimental data set (see Fig. S4.3 below which shows the dorso-ventral distribution of these axons).

Table 2. Performance of the model for different degrees of parameter variation

case	value of *index*				quality of optimization		
#	alpha	gamma	mu	ybar	Q(5%)	Q(10%)	Q(20%)
1	-1	-1	-1	-1	8.76	22.05	91.55
2	-1	-1	-1	0	4.31	4.07	4.22
3	-1	-1	-1	1	5.77	9.55	21.39
4	-1	-1	0	-1	5.59	10.02	39.90
5	-1	-1	0	0	4.08	5.73	11.32
6	-1	-1	0	1	7.34	15.99	44.83
7	-1	-1	1	-1	4.65	5.25	16.77
8	-1	-1	1	0	5.47	9.82	25.12
9	-1	-1	1	1	10.00	24.63	70.43
10	-1	0	-1	-1	13.49	53.28	288.08
11	-1	0	-1	0	5.83	9.38	32.76
12	-1	0	-1	1	4.44	4.83	5.91
13	-1	0	0	-1	10.02	27.67	198.88
14	-1	0	0	0	4.51	4.63	5.63
15	-1	0	0	1	5.61	8.70	19.88
16	-1	0	1	-1	6.48	15.23	129.85
17	-1	0	1	0	4.07	5.02	8.45
18	-1	0	1	1	7.28	14.55	38.73
19	-1	1	-1	-1	23.36	121.69	577.93
20	-1	1	-1	0	10.12	26.09	155.43
21	-1	1	-1	1	4.69	5.91	11.38
22	-1	1	0	-1	18.13	78.40	354.82
23	-1	1	0	0	5.91	12.17	46.41
24	-1	1	0	1	4.32	4.54	6.71
25	-1	1	1	-1	11.36	37.27	249.86
26	-1	1	1	0	4.81	5.04	10.61
27	-1	1	1	1	5.01	8.40	19.29
28	0	-1	-1	-1	7.26	16.27	50.14
29	0	-1	-1	0	4.38	4.64	5.65
30	0	-1	-1	1	5.84	10.10	22.15
31	0	-1	0	-1	4.98	8.09	17.15
32	0	-1	0	0	4.46	6.25	12.36
33	0	-1	0	1	7.52	16.93	44.34
34	0	-1	1	-1	4.34	3.86	6.11
35	0	-1	1	0	5.62	9.90	25.84
36	0	-1	1	1	9.89	24.07	68.00
37	0	0	-1	-1	12.48	35.72	205.52

case	value of *index*				quality of optimization		
#	alpha	gamma	mu	ybar	Q(5%)	Q(10%)	Q(20%)
38	0	0	-1	0	5.75	8.08	19.18
39	0	0	-1	1	4.58	5.65	7.76
40	0	0	0	-1	9.62	21.85	103.94
41	0	0	0	0	4.20	4.32	4.18
42	0	0	0	1	5.50	9.26	20.19
43	0	0	1	-1	5.91	10.70	59.80
44	0	0	1	0	4.16	4.84	8.40
45	0	0	1	1	7.09	14.68	38.82
46	0	1	-1	-1	20.80	80.36	380.80
47	0	1	-1	0	9.76	18.97	103.05
48	0	1	-1	1	4.65	5.27	8.91
49	0	1	0	-1	12.68	43.65	274.92
50	0	1	0	0	6.10	8.44	24.03
51	0	1	0	1	4.32	5.12	7.74
52	0	1	1	-1	9.19	29.89	184.43
53	0	1	1	0	4.35	4.46	4.94
54	0	1	1	1	5.21	8.84	19.37
55	1	-1	-1	-1	7.36	13.29	30.91
56	1	-1	-1	0	4.46	5.74	8.57
57	1	-1	-1	1	6.07	11.30	23.76
58	1	-1	0	-1	5.37	6.55	12.55
59	1	-1	0	0	5.02	6.99	13.67
60	1	-1	0	1	8.13	16.88	43.92
61	1	-1	1	-1	4.34	4.03	5.01
62	1	-1	1	0	6.11	10.57	26.03
63	1	-1	1	1	10.36	24.55	67.62
64	1	0	-1	-1	11.24	32.00	111.02
65	1	0	-1	0	5.54	7.94	15.76
66	1	0	-1	1	4.96	6.35	9.56
67	1	0	0	-1	7.66	16.04	56.80
68	1	0	0	0	4.15	4.78	5.52
69	1	0	0	1	5.68	9.63	22.18
70	1	0	1	-1	5.38	8.91	26.40
71	1	0	1	0	4.43	5.48	9.67
72	1	0	1	1	7.31	15.35	37.81
73	1	1	-1	-1	16.99	59.76	291.09
74	1	1	-1	0	8.30	17.50	58.05
75	1	1	-1	1	4.78	5.98	9.35
76	1	1	0	-1	12.30	35.43	180.90
77	1	1	0	0	5.49	7.48	14.64
78	1	1	0	1	4.69	5.56	8.48
79	1	1	1	-1	8.56	19.83	112.91
80	1	1	1	0	4.26	4.48	4.64
81	1	1	1	1	5.57	9.28	20.45

F. Optimization results for each neuron type and examples of generated model axons compared with measured axons

The following 10 pages contain three figures for each neuron type. The first figure shows the test histograms and the caption of this figure indicates the quality of the optimization. The second figure shows model axons (upper panel) and experimental axons (lower panel); the caption gives both experimental and model axon tortuosities. The third figure shows distributions of dorso-ventral coordinates of experimental and generated axons.

1. Test results for aIN descending

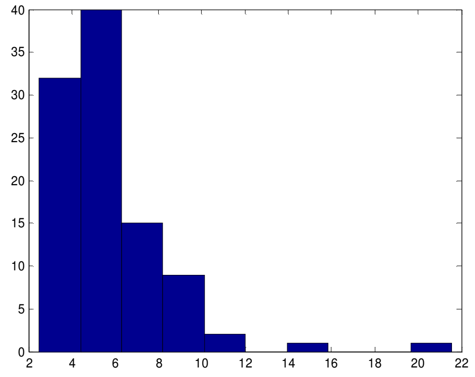

Fig. S1.1 Test of optimization quality: GOOD.
Distribution of averaged cost function values in 100 trials. Each trial includes generation of 300 axons.
Horizontal axis shows cost function value allocated in bins of the histogram and vertical axis shows percentage for the bin.

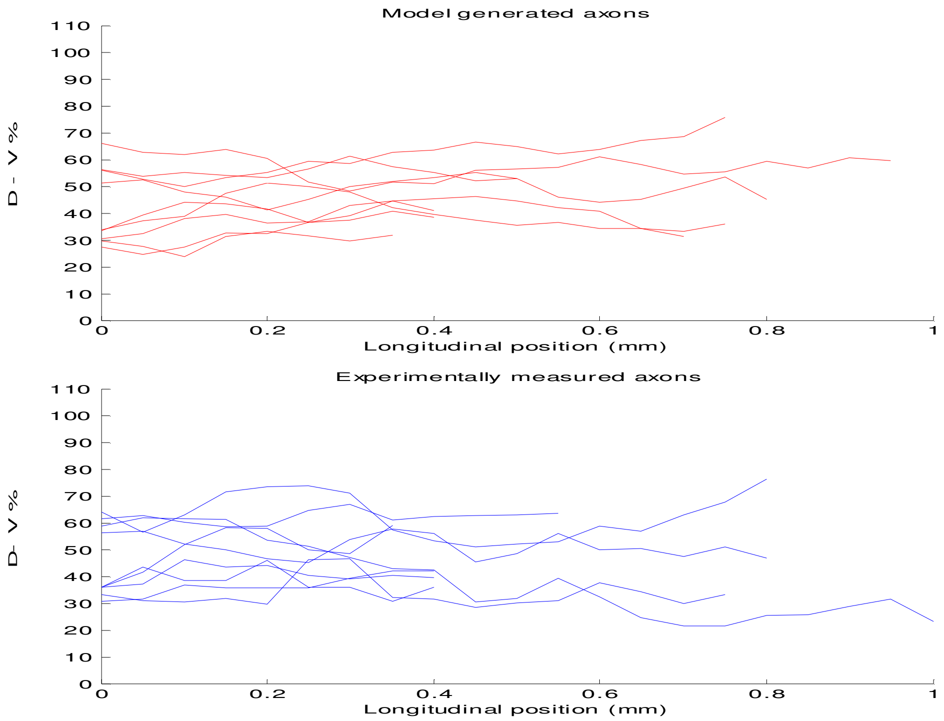

Fig. S1.2 An example of axons generated by model (upper panel in red) and experimentally measured axons (lower panel in blue).
Model tortuosity: 1.002. Experimental tortuosity: 1.005

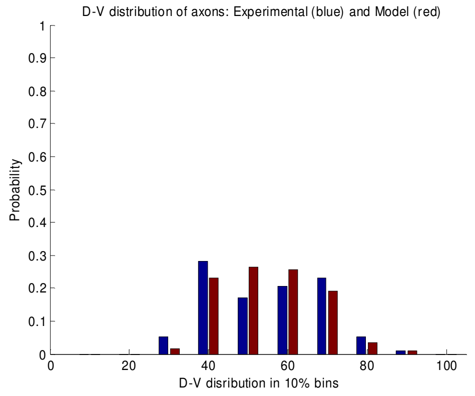

Fig. S1.3 Comparing the dorso-ventral distributions of model axons (blue) and experimental axons (red).

2. Test results for aIN ascending

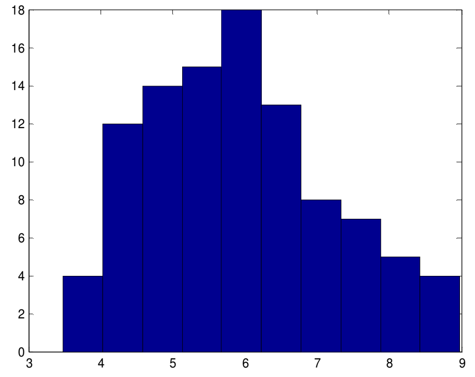

Fig. S2.1 Test of optimization quality: GOOD.
Distribution of averaged cost function values in 100 trials. Each trial includes generation of 300 axons
Horizontal axis shows cost function value allocated in bins of the histogram and vertical axis shows percentage for the bin.

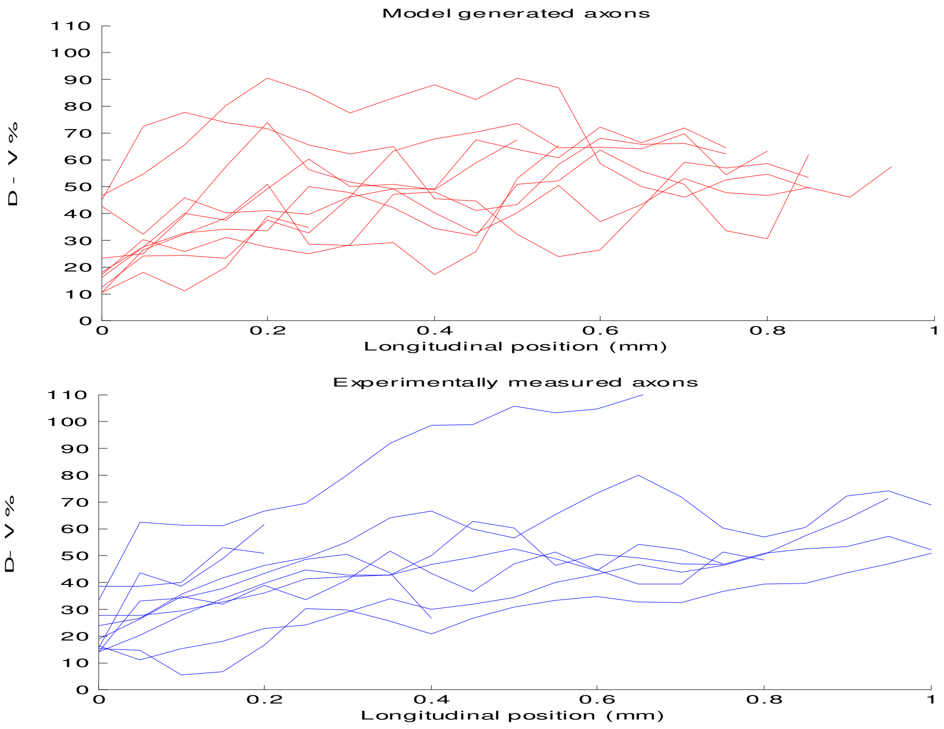

Fig. S2.2 An example of axons generated by model (upper panel in red) and experimentally measured axons (lower panel in blue).
Model tortuosity: 1.02. Experimental tortuosity: 1.009

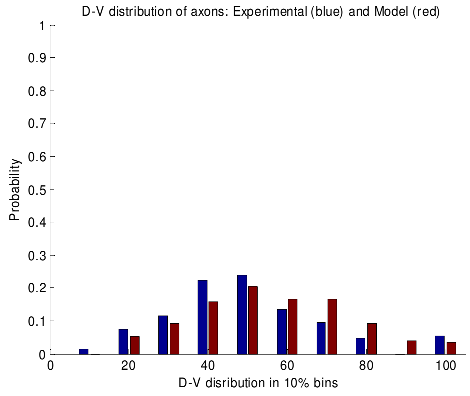

Fig. S2.3 Comparing the dorso-ventral distributions of model axons (blue) and experimental axons (red).

3. Test results for cIN descending

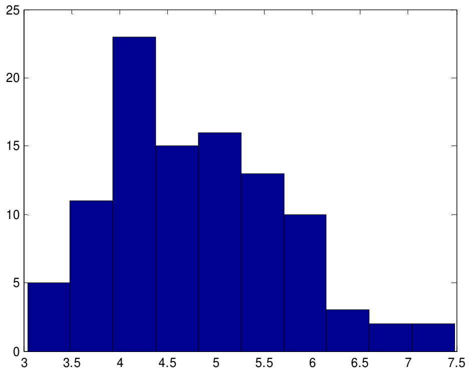

Fig. S3.1 Test of optimization quality: GOOD.
Distribution of averaged cost function values in 100 trials. Each trial includes generation of 300 axons
Horizontal axis shows cost function value allocated in bins of the histogram and vertical axis shows percentage for the bin.

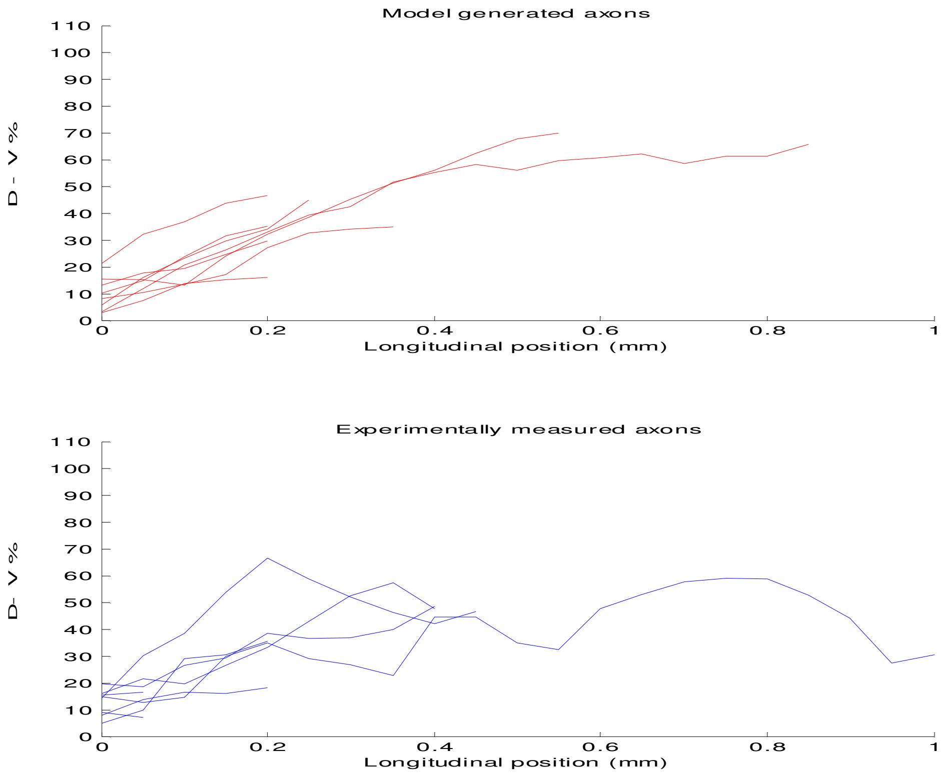

Fig. S3.2 An example of axons generated by model (upper panel in red) and experimentally measured axons (lower panel in blue).
Model tortuosity: 1.001. Experimental tortuosity: 1.006

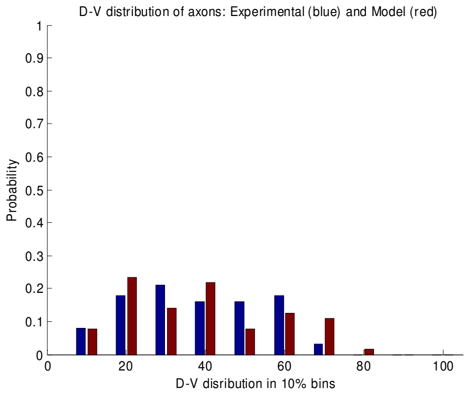

Fig. S3.3 Comparing the dorso-ventral distributions of model axons (blue) and experimental axons (red).

4. Test results for cIN ascending

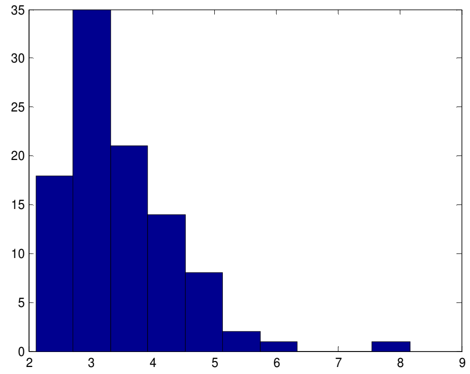

Fig. S4.1 Test of optimization quality: GOOD.
Distribution of averaged cost function values in 100 trials. Each trial includes generation of 300 axons.
Horizontal axis shows cost function value allocated in bins of the histogram and vertical axis shows percentage for the bin.

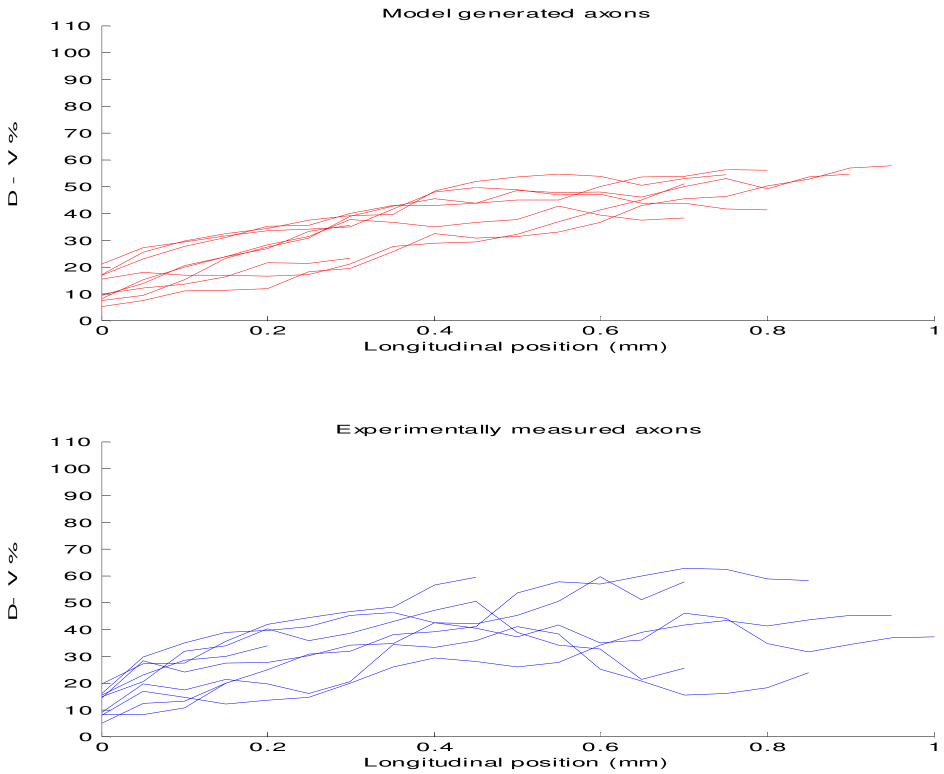

Fig. S4.2 An example of axons generated by model (upper panel in red) and experimentally measured axons (lower panel in blue).
Model tortuosity: 1.001. Experimental tortuosity: 1.004

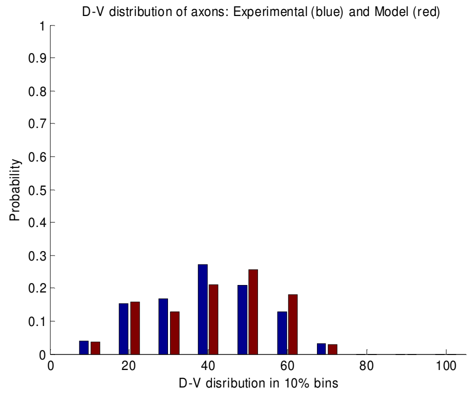

Fig. S4.3 Comparing the dorso-ventral distributions of model axons (blue) and experimental axons (red).

5. Test results for dIN descending

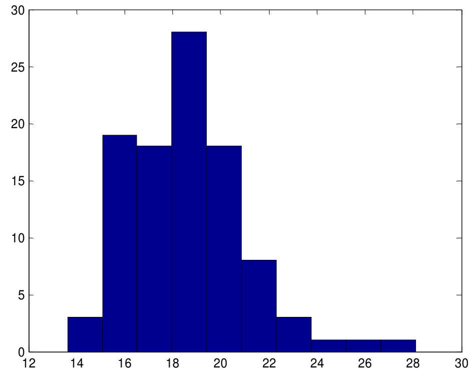

Fig. S5.1 Test of optimization quality: POOR.
Distribution of averaged cost function values in 100 trials. Each trial includes generation of 300 axons.
Horizontal axis shows cost function value allocated in bins of the histogram and vertical axis shows percentage for the bin.

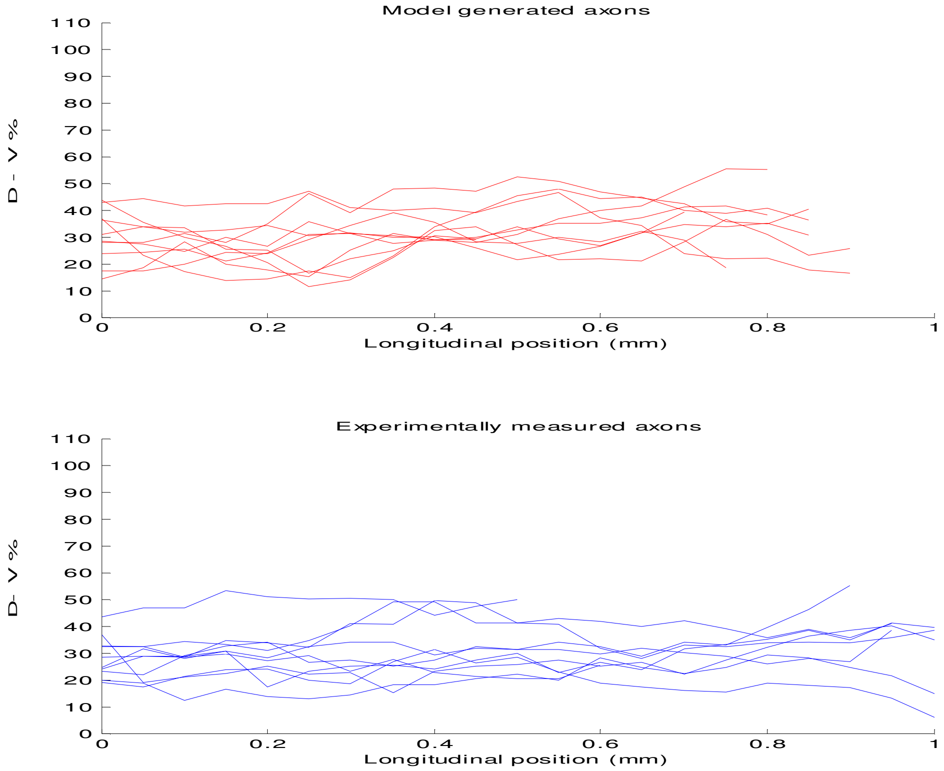

Fig. S5.2 An example of axons generated by model (upper panel in red) and experimentally measured axons (lower panel in blue).
Model tortuosity: 1.004. Experimental tortuosity: 1.003

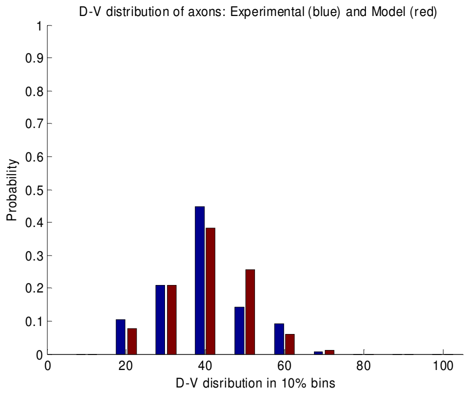

Fig. S5.3 Comparing the dorso-ventral distributions of model axons (blue) and experimental axons (red).

6. Test results for RB descending

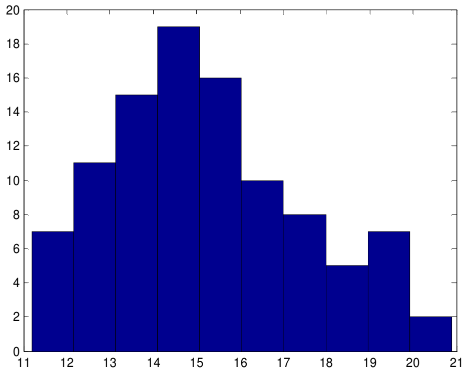

Fig. S6.1 Test of optimization quality: POOR.
Distribution of averaged cost function values in 100 trials. Each trial includes generation of 300 axons.
Horizontal axis shows cost function value allocated in bins of the histogram and vertical axis shows percentage for the bin.

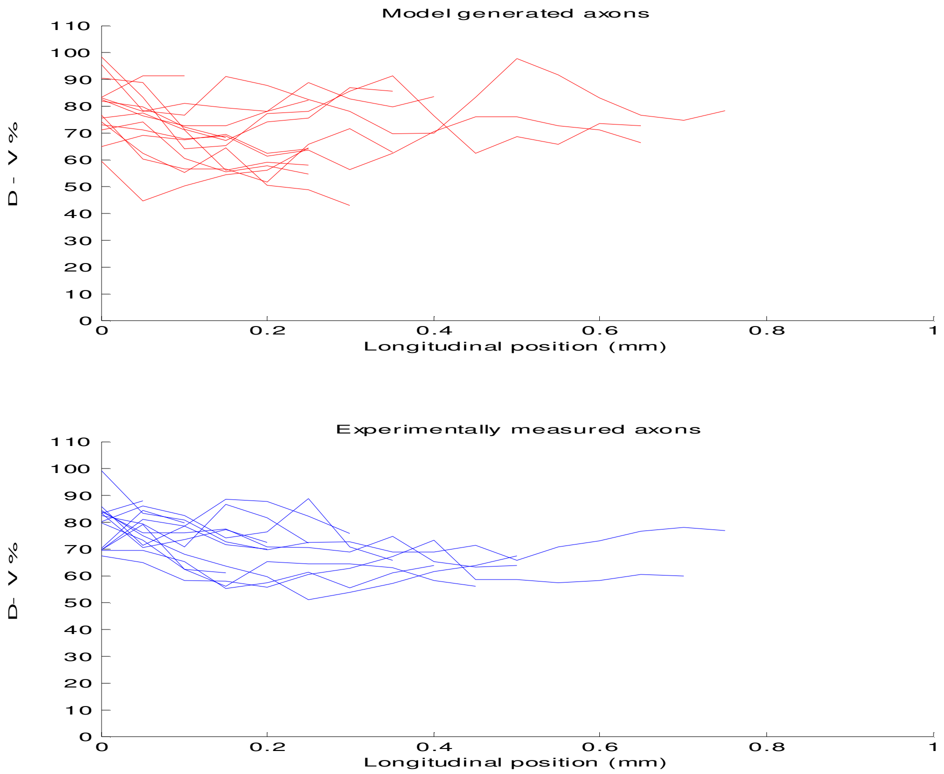

Fig. S6.2 An example of axons generated by model (upper panel in red) and experimentally measured axons (lower panel in blue).
Model tortuosity: 1.009. Experimental tortuosity: 1.009

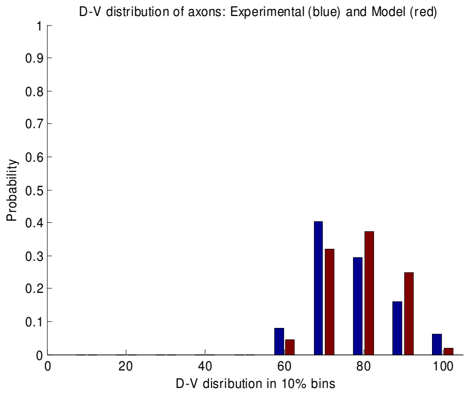

Fig. S6.3 Comparing the dorso-ventral distributions of model axons (blue) and experimental axons (red).

7. Test results for RB ascending

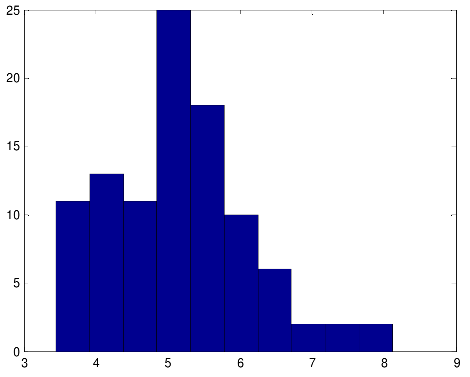

Fig. S7.1 Test of optimization quality: GOOD.
Distribution of averaged cost function values in 100 trials. Each trial includes generation of 300 axons
Horizontal axis shows cost function value allocated in bins of the histogram and vertical axis shows percentage for the bin.

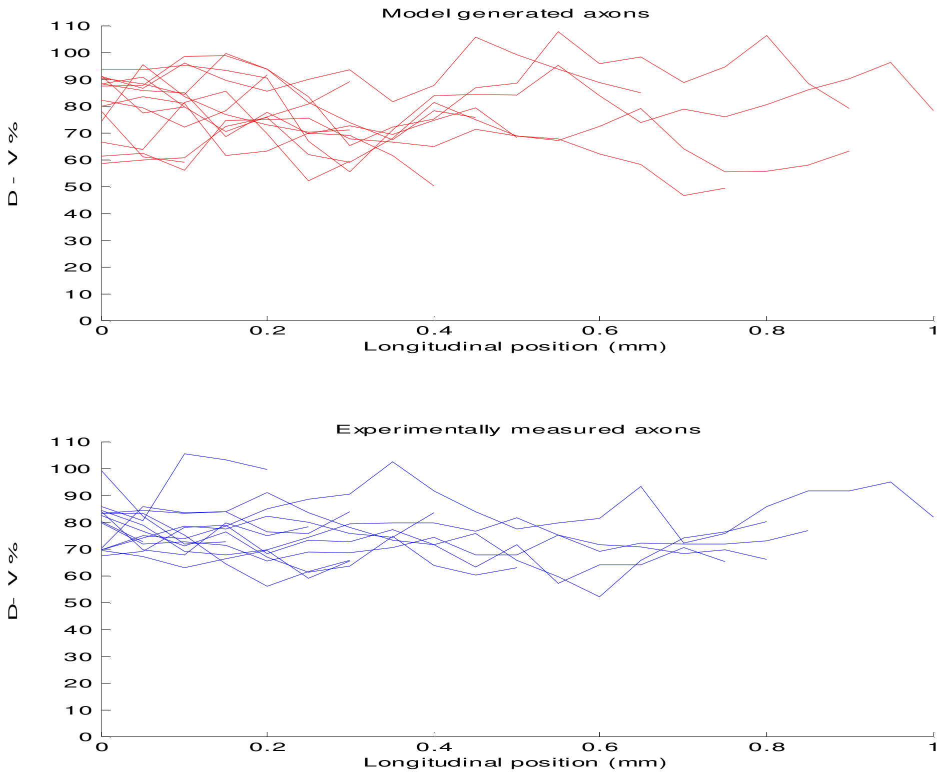

Fig. S7.2 An example of axons generated by model (upper panel in red) and experimentally measured axons (lower panel in blue).
Model tortuosity: 1.01. Experimental tortuosity: 1.009

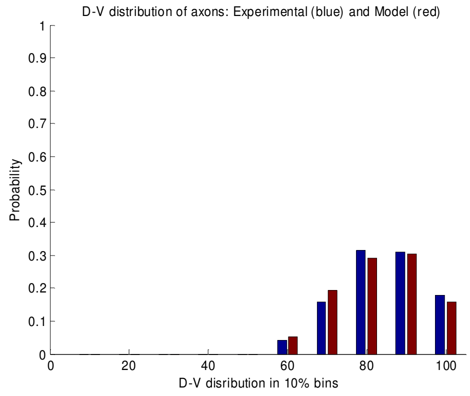

Fig. S7.3 Comparing the dorso-ventral distributions of model axons (blue) and experimental axons (red).

8. Test results for dlc descending

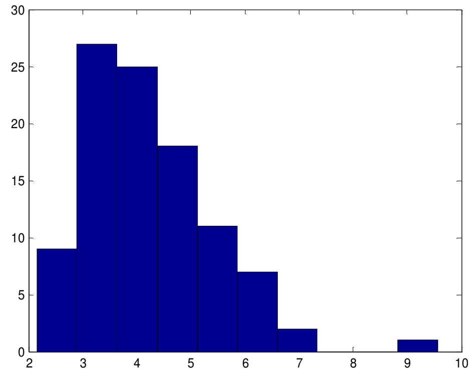

Fig. S8.1 Test of optimization quality: GOOD.
Distribution of averaged cost function values in 100 trials. Each trial includes generation of 300 axons.
Horizontal axis shows cost function value allocated in bins of the histogram and vertical axis shows percentage for the bin.

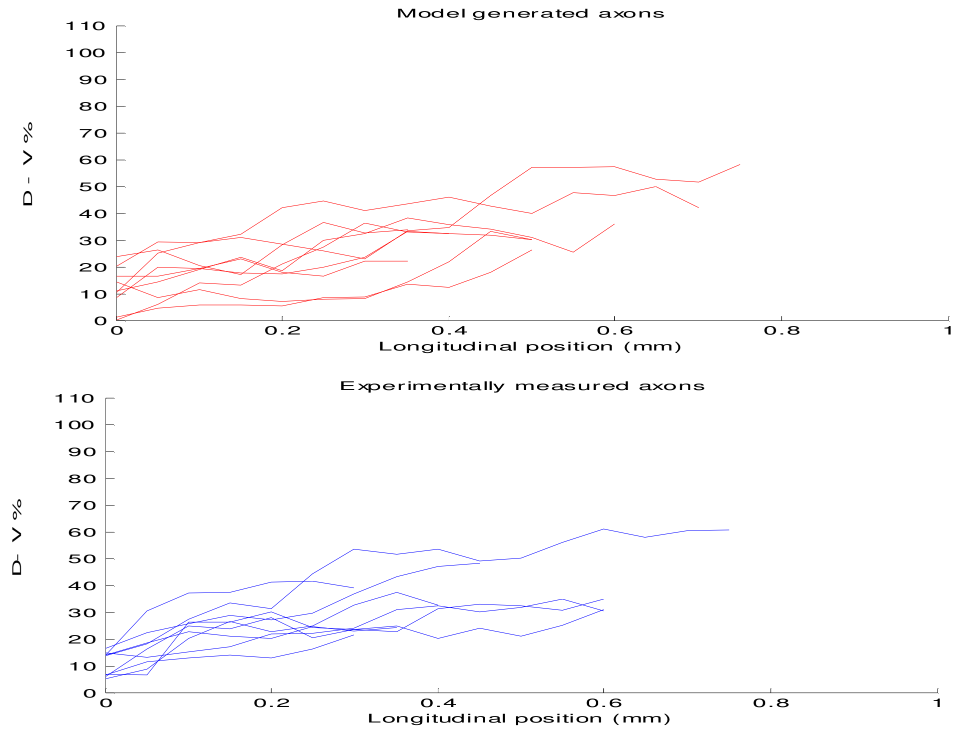

Fig. S8.2 An example of axons generated by model (upper panel in red) and experimentally measured axons (lower panel in blue).
Model tortuosity: 1.002. Experimental tortuosity: 1.005

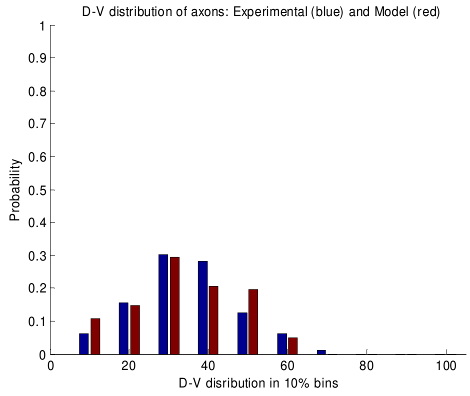

Fig. S8.3 Comparing the dorso-ventral distributions of model axons (blue) and experimental axons (red).

9. Test results for dlc ascending

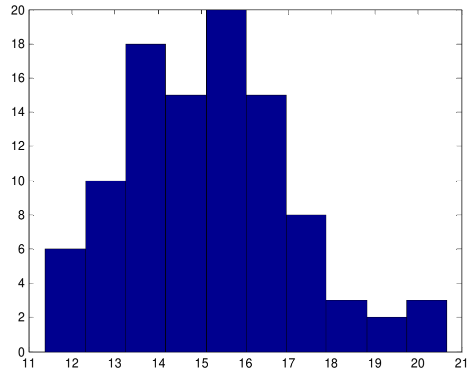

Fig. S9.1 Test of optimization quality: POOR.
Distribution of averaged cost function values in 100 trials. Each trial includes generation of 300 axons.
Horizontal axis shows cost function value allocated in bins of the histogram and vertical axis shows percentage for the bin.

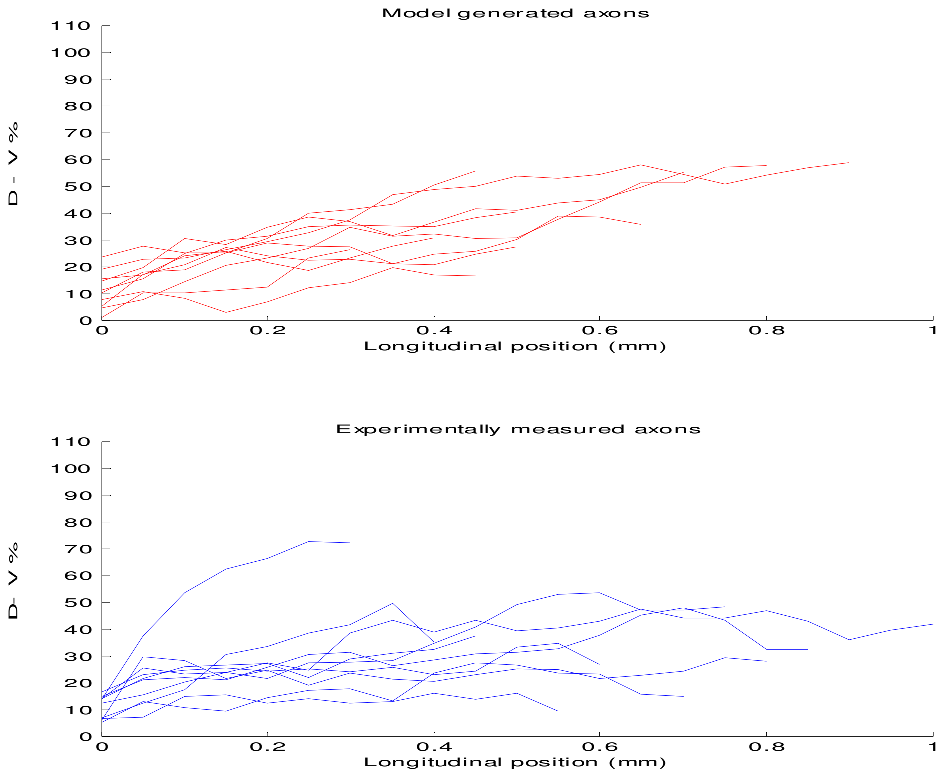

Fig. S9.2 An example of axons generated by model (upper panel in red) and experimentally measured axons (lower panel in blue).
Model tortuosity: 1.003. Experimental tortuosity: 1.006

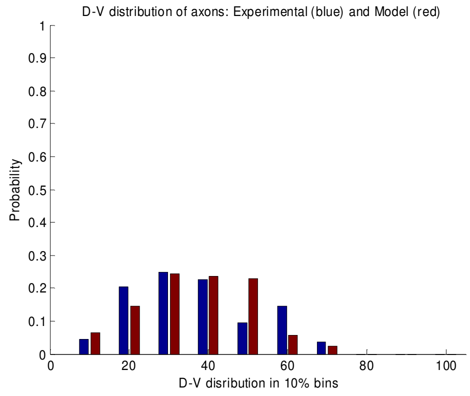

Fig. S9.3 Comparing the dorso-ventral distributions of model axons (blue) and experimental axons (red).

10. Testing results for mn descending

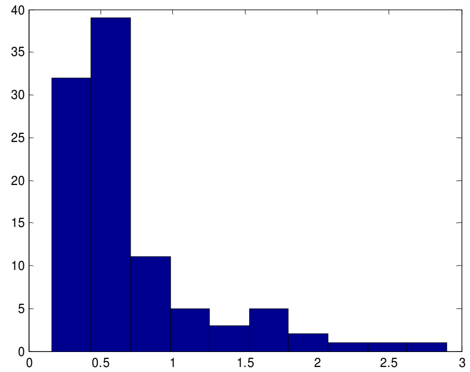

Fig. S10.1 Test of optimization quality: VERY GOOD.
Distribution of averaged cost function values in 100 trials. Each trial includes generation of 300 axons.
Horizontal axis shows cost function value allocated in bins of the histogram and vertical axis shows percentage for the bin.

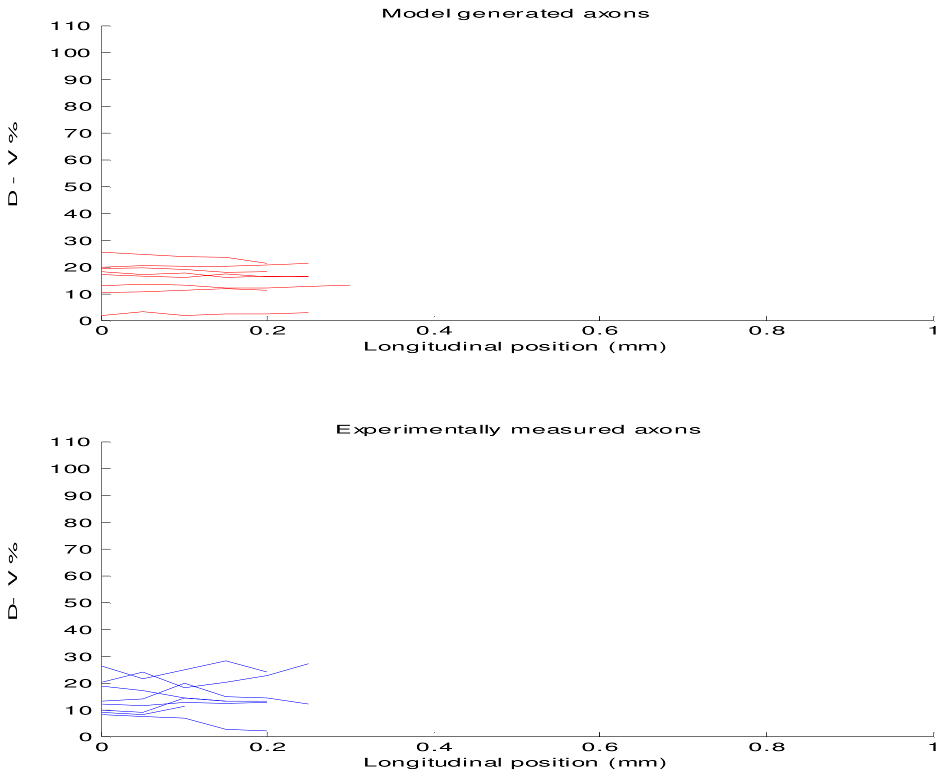

Fig. S10.2 An example of axons generated by model (upper panel in red) and experimentally measured axons (lower panel in blue).
Model tortuosity: 1.0001. Experimental tortuosity: 1.001

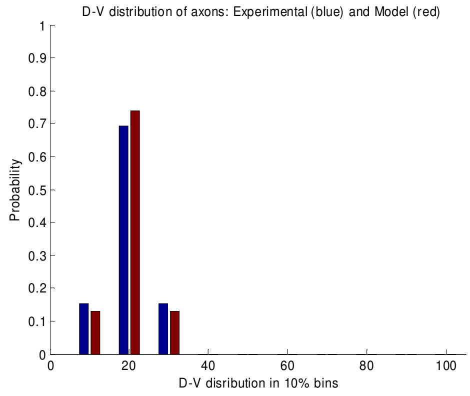

Fig. S10.3 Comparing the dorso-ventral distributions of model axons (blue) and experimental axons (red).

3) Network modelling

Table of maximum conductances (in nS) for synapses in network model.

		probability model	
**source**	**target**	**AMPA**	**NMDA**
**RB**	**dlc**	7,03	0,14
	**dla**	3,40	0,16
	**aIN**	0,23	0,11
	**cIN**	0,23	0,11
	**dIN**	0,16	0,08
	**MN**	0,30	0,14
**dlc**	**CPG**	2,16	1,32
	**aIN**	1,76	1,39
	**cIN**	1,44	2,74
	**dIN**	4,00	0,81
	**MN**	1,43	0,34
**dla**	**CPG**	2,07	1,36
	**aIN**	3,87	1,35
	**cIN**	1,03	2,31
	**dIN**	2,25	1,47
	**MN**	1,13	0,31
**dIN**	**aIN**	1,94	0,67
	**cIN**	8,41	2,79
	**dIN**	4,00	6,66
	**MN**	4,47	1,27
		**Inhibition**	
**cIN**	**aIN**	2,93	
	**cIN**	1,98	
	**dIN**	7,29	
	**MN**	3,67	
**aIN**	**cIN**	0,78	
	**dIN**	1,26	
	**MN**	1,22	
	**dlc**	4,79	
	**dla**	4,38	

References

Goodhill GJ, Gu M, Urbach JS (2004) Predicting axonal response to molecular gradients with a computational model of filopodial dynamics. Neural Computation 16:2221 - 2243.

Krottje JK, van Ooyen A (2007) A Mathematical Framework for Modeling Axon Guidance. Bulletin of Mathematical Biology 69:3 - 31.

Lagarias JC, Reeds JA, Wright MH, Wright PE (1998) Convergence Properties of the Nelder-Mead Simplex Method in Low Dimensions. SIAM Journal of Optimization 9:112 - 147.
